# FineSplice, enhanced splice junction detection and quantification: a novel pipeline based on the assessment of diverse RNA-Seq alignment solutions

**DOI:** 10.1093/nar/gku166

**Published:** 2014-02-25

**Authors:** Alberto Gatto, Carlos Torroja-Fungairiño, Francesco Mazzarotto, Stuart A. Cook, Paul J. R. Barton, Fátima Sánchez-Cabo, Enrique Lara-Pezzi

**Affiliations:** ^1^Cardiovascular Development and Repair Department, Centro Nacional de Investigaciones Cardiovasculares, Madrid, 28029, Spain, ^2^Bioinformatics Unit, Centro Nacional de Investigaciones Cardiovasculares, Madrid, 28029, Spain, ^3^National Heart and Lung Institute, Imperial College London, London SW7 2AZ, UK, ^4^Cardiovascular Biomedical Research Unit, NIHR Royal Brompton and Harefield NHS Foundation Trust, London SW3 6NP, UK, ^5^Department of Cardiology, National Heart Centre Singapore, Singapore 168752, Singapore and ^6^Cardiovascular and Metabolic Disorders Program, Duke-NUS Graduate Medical School, Singapore 169857, Singapore

## Abstract

Alternative splicing is the main mechanism governing protein diversity. The recent developments in RNA-Seq technology have enabled the study of the global impact and regulation of this biological process. However, the lack of standardized protocols constitutes a major bottleneck in the analysis of alternative splicing. This is particularly important for the identification of exon–exon junctions, which is a critical step in any analysis workflow. Here we performed a systematic benchmarking of alignment tools to dissect the impact of design and method on the mapping, detection and quantification of splice junctions from multi-exon reads. Accordingly, we devised a novel pipeline based on TopHat2 combined with a splice junction detection algorithm, which we have named FineSplice. FineSplice allows effective elimination of spurious junction hits arising from artefactual alignments, achieving up to 99% precision in both real and simulated data sets and yielding superior F_1_ scores under most tested conditions. The proposed strategy conjugates an efficient mapping solution with a semi-supervised anomaly detection scheme to filter out false positives and allows reliable estimation of expressed junctions from the alignment output. Ultimately this provides more accurate information to identify meaningful splicing patterns. FineSplice is freely available at https://sourceforge.net/p/finesplice/.

## INTRODUCTION

Of the many actors involved in mRNA regulation, alternative splicing (AS) plays the lead role in shaping the post-transcriptional landscape. Through differential exon inclusion, intron retention and alternative splice site usage, AS allows for the generation of multiple transcript and protein isoforms from a single gene, bridging the gap between the great structural and functional diversity of the eukaryotic proteome and the relatively small amount of coding genes (1). AS constitutes a flexible, conserved and dynamic layer of regulation, affecting the vast majority of multi-exon genes (2), often in a tissue-specific fashion (3,4), and modulating phenotypic changes with wide-ranging implications in morphogenesis (5,6), evolution (7,8) and disease (9–12). Despite the extensive evidence about its functional relevance, the global impact and regulation of AS are far from being completely understood.

In recent years, the advent of RNA-Seq has boosted research in the AS field and is paving the way to a genome-wide understanding of its regulatory mechanisms and effects in different biological contexts (2,13). Besides allowing for the comparison of gene expression changes, RNA-Seq makes it possible to identify novel isoforms, assess relative transcript abundances and detect alternative exon and splice site usage (14–16). Using next-generation sequencing, knowledge is being accumulated at an incredibly fast pace, nourishing the expectations of an integrated splicing code that would allow to predict the occurrence and impact of specific splicing patterns under different conditions (17,18). Whereas computational solutions to study gene expression are relatively well established, best practices in AS data analysis remain a largely open-ended issue. A large, and constantly increasing, number of algorithms address the analysis of AS at different levels (15,19–21), providing various, often disparate, solutions to the many challenges posed by AS in terms of mapping, normalization and statistical analysis of RNA-Seq data (22–30). The lack of an integrated framework and standardized guidelines constitutes nonetheless a major bottleneck, and the suitability of different methods, depending on the aim of the study and the experimental set-up, is unclear.

Here we address the problem at its fundamental level: the definition of a reliable set of expressed splice junctions in a typical context where the full set of transcripts is not entirely known. Reads that overlap multiple exons represent, in this respect, the most basic unambiguous piece of information retrievable from an RNA-Seq experiment with a direct relevance to AS (31,32). From this perspective, the problem comes down to three main objectives, all of which basically rely on the effectiveness of the alignment process: (i) split-read mapping and *de novo* splice junction discovery, (ii) detection of expressed junctions from reliable alignment hits and (iii) quantification of expression levels by read counting. This makes the analysis conceptually straightforward, and allows for the strengths and drawbacks of the different methods to be clearly dissected. For each of these tasks, we evaluated the performance of five alignment algorithms on synthetic data sets. Based on the results, we propose an integrated pipeline to conjugate the needs of a reliable detection with those of an accurate mapping and expression estimation. To this end, we developed an ad hoc post-processing strategy, FineSplice, to filter out false-positive hits via a semi-supervised anomaly detection method. We tested FineSplice detection performance under all simulation settings and on publicly available experimental RNA-Seq data. The suggested pipeline provides a simple effective solution to address the analysis of RNA-Seq data at the splice junction level, achieving superior results in terms of detection precision while attaining high mapping and quantification accuracy.

## MATERIALS AND METHODS

### Simulation of RNA-Seq experiments

A total of 10 random data sets for 12 different experimental set-ups were generated using the Flux Simulator (33) pipeline (version 1.2), based on the GRCh37.p8 assembly of the human genome and Ensembl genebuild (release 69) annotation (34). Each combination of the following parameters was used to generate a data set: 50 and 76 bp read length, 8M, 20M and 40M reads sequencing depth, single-end and paired-end library. Following the procedure in the documentation, a custom error model at 50 bp read length was produced using in-house RNA-Seq data (Supplementary Figure S1). The sequencing run was deposited in the NCBI Sequence Read Archive, with accession number SRR1105576. Default parameters were used for all other options. For each simulated data set, 10% of the exons at each expression decile were removed from the original annotation to evaluate the *de novo* splice junction detection capability and the impact of novel and misannotated features.

### Alignment algorithm benchmarking

The following alignment algorithms have been tested: TopHat2 version 2.0.6 (35), GSNAP version 2012-12-20 (36,37), STAR version 2.2.0 (38), OLego version 1.08 (39) and SOAPsplice version 1.9 (40). All aligners were run with default parameters. For paired-end data, whereby required, the insert size was empirically determined from uniquely mapping, perfect matching pairs via a preliminary alignment with Bowtie version 0.12.9 (41) and supplied to the algorithm. Except for SOAPsplice, which is an *ab initio* alignment method, input annotations were constructed to comply the required format of each aligner. Site-level input files were produced for GSNAP, as suggested in the documentation.

### Performance evaluation

For both known and novel exon junctions, the mapping performance was evaluated in terms of percentage of uniquely mapped reads and positive predictive value over unique alignments at base pair resolution. Nucleotides mapped to the wrong genomic location were regarded as false positives, correctly aligned nucleotides as true positives. The junction detection performance was assessed in terms of sensitivity and positive predictive value based on unique gapped alignments reported with an N operation in the CIGAR string. Expressed junctions in the simulated data spanned by at least one read in the alignment were considered true positives. Expressed junctions with no unique hits were regarded as false negatives and junctions spanned by at least one read in the alignment but not present in the simulated data as false positives. Over all detectable junctions, quantification accuracy was assessed in terms of absolute difference between true counts and alignment counts (number of uniquely mapped reads spanning a junction). For true positive hits, the absolute difference was as well computed relative to the true expression value. Plots were generated using ggplot2 (version 0.9.3.1) R package (42).

### The FineSplice pipeline

*Step 1. Align with TopHat2*. Transcriptome alignment with de novo splice junction discovery is performed using TopHat2 with available annotations for known transcript isoforms.

*Step 2. Compute the set of split-read overhangs across each junction*. For each uniquely mapping read 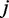 spanning a given junction 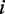, its overhang 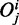 is defined as the shortest overlapping segment of the read across the junction, i.e.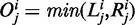, where 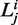 and 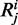 represent the length of the left and right arm of the read across the junction. Each junction is hence represented by a set 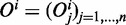 of 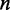 split-reads overhangs. Under the assumption of random cDNA fragmentation, all overhangs are taken to be equally likely and 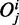 hence assumed to follow a discrete uniform distribution 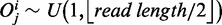.

*Step 3. Define a subset of potential false positives*. For each junction, if (i) a single mismatch is present and (ii) none out of 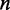 reads is found with an overhang greater than the first mismatching position, the following probability is considered:

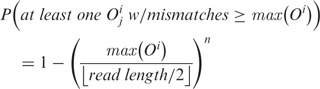



If >0.99, the junction 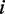 is deemed as a potential false positive and labeled 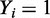. Splice junctions with no matching overhang are labelled as well as potential false positives. The remaining total of detectable junctions is assumed to mostly comprise valid spliced alignments and assigned the class label 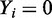.

*Step 4. Construct feature vectors*. For all possible overhang values 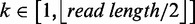, let 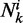 be the number of reads with an overhang larger than 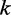 after trimming mismatching overhangs at the first mismatch position. For each junction 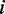, a feature vector 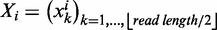 is constructed based on the log_2_ deviation of observed counts from expected at each position relative to the splice site, i.e.

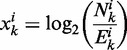

where 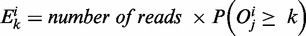 and 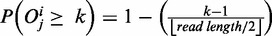.

*Step 5. Fit a logistic regression model*. Following step 3 and 4, each junction is represented by a class label 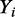 and a feature vector 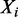. A L1-regularized logistic regression model is therefore fitted over the whole set of junctions.

*Step 6. Discard spurious alignments based on posterior probability*. For each junction 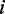, the posterior probability of belonging to the false-positive class is computed: if 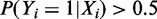, the junction is deemed as a false positive and discarded.

*Step 7. Rescue multiple mapping reads*. Reads mapped to multiple splice sites for which a unique hit is recovered after filtering are allocated to the accepted junction.

FineSplice depends on pysam (version 0.7.4), scipy (version 0.7.2) and numpy (version 1.7.1) modules for BAM file parsing and scientific computing. The logistic regression model relies on scikit-learn (version 0.13.1) implementation (43), based on the LIBLINEAR library (44,45).

### FineSplice testing in simulated and real data

FineSplice improvement in splice junction detection over TopHat2 was assessed in synthetic data, under all simulation settings, allowing for multiple alignment options. Additional TopHat2 alignments were performed enabling the realignment option for ambiguously mapping multi-exon reads, with a cut-off of either one or two mismatches in the segment alignment step. FineSplice performance was further compared with TrueSight version 0.06 (46), a recently published *ab initio* alignment method using logistic regression to enhance junction mapping. TrueSight was run with default parameters, and splice junctions, together with the associated score (posterior probability), were retrieved from the corresponding output. Precision and sensitivity were computed as described above, both in the default setting and at increasing thresholds for the posterior probability estimates of FineSplice and TrueSight.

The splice junction detection performance was moreover evaluated on real data from publicly available RNA-Seq experiments in human (two data sets, high-quality or low-quality reads) and pig (poorly annotated transcriptome). The high-quality human data set comprises three high-depth paired-end sequencing runs at 76 bp read length (Supplementary Figure S2, SRA Experiment SRX084679). The low-quality human data set comprises two low-depth paired-end sequencing runs at 45 bp read length, exhibiting a high per base error rate (Supplementary Figure S3, SRA Experiment SRX011546). The pig data set comprises three sequencing runs at 51 bp read length, single-end (Supplementary Figure S4, SRA Experiments SRX242929, SRX242930 and SRX242931). Raw data were downloaded from the NCBI Short Read Archive (SRA) and converted to FASTQ with the SRA Toolkit. The alignment was carried out with the five benchmarked algorithms plus TrueSight, as described above, but using the full transcript annotation (whereby possible). In the absence of ground truth, the detection performance was evaluated by computing pseudo sensitivity and pseudo precision metrics (38,47), along with the corresponding F_1_ score, and by evaluating the mean read counts and the consensus across all alignments for all the junctions detected by each algorithm. Splice junctions were designated as pseudo true (i.e. effectively expressed) based on read counts and consensus among all methods, by deeming as effectively expressed those with a median read count across all methods >0. The distribution of read counts at each overhang position over all alignments was further assessed for all junctions accepted and discarded by FineSplice.

## RESULTS

### Splice junction mapping performance

The crucial and most demanding step in a typical AS analysis workflow is aligning reads that span exon–exon junctions in an effective manner. To evaluate the impact of experimental design, alignment method and prior knowledge on the mapping performance, we systematically compared the behaviour of different algorithms under varying simulated set-ups (different read lengths, sequencing depths and library preparation protocols), across 10 random data sets per experimental condition. We generated sampled transcript annotations via a random exclusion of exon features at different expression deciles for each experiment. We evaluated a selection of five alignment algorithms: TopHat2, GSNAP, STAR, SOAPsplice and OLego. These aligners offer different approaches to mapping (exon-first, seed-and-extend and multi-seed), indexing (hash table and FM-index), annotation use (transcript-based, intron-based and *ab initio*) and *de novo* splice site prediction [see (21) for a comprehensive review]. The benchmarked methods mostly take advantage of available annotations either at the transcriptome level (full-length transcripts or known combinations of donors and acceptors e.g. TopHat2 and GSNAP) or at the intron level (set of donor–acceptor pairs e.g. OLego, STAR), except for SOAPsplice, which is geared towards *ab initio* detection of junction spanning reads.

**Figure 1.**
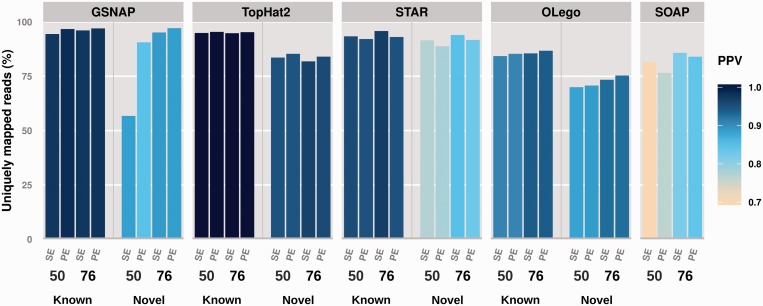
TopHat2 provides the best trade-off in terms of alignment precision and percentage of uniquely mapped reads, for both known and novel junctions. The percentage of uniquely mapped reads (bar chart, y-axis) and the positive predictive value (ratio of correctly aligned nucleotides, colour coded), averaged over 10 simulated data sets per experimental condition, are shown for each alignment method on a separate panel. With the exception of SOAPsplice, which exclusively aligns reads *ab initio*, left and right sides of each panel correspond, respectively, to reads spanning known and novel junctions (i.e. randomly included or excluded from the input annotation). Each bar corresponds to a different simulation set-up: 50 or 76 bp read length, single-end (SE) or paired-end (PE) library, at 20 M reads sequencing depth.

Figure 1 summarizes the average performance across the 10 simulated data sets per experimental setting at 20 M reads sequencing depth. Results at 8 M and 40 M reads sequencing depth are shown in Supplementary Figure S5. Under all tested conditions, for both known and novel junctions, TopHat2 appears to outperform other methods in terms of mapping precision (positive predictive value, colour coded) while attaining, at the same time, a high percentage of uniquely mapping reads. Overall, TopHat2 provides the best trade-off in terms of mapping performance, even when other algorithms exhibit slightly higher percentages of unique hits (e.g. STAR or GSNAP). With the exception of sequencing depth, which does not appear to substantially affect the results, the impact of experimental design is mostly aligner-dependent. TopHat2 shows small but consistent improvements with paired-end data (whereas STAR performs slightly worse), OLego and GSNAP achieve better results with increasing read lengths and paired-end information, as well as SOAPsplice (mostly in terms of positive predictive value). Across the 10 data sets generated for each simulation set-up, the alignment performance was rather consistent, with low standard deviations both in the case of junction-spanning reads (Supplementary Tables S1 and S2) and considering also exon mapping reads (Supplementary Table S3).

### Splice junction detection performance

**Figure 2.**
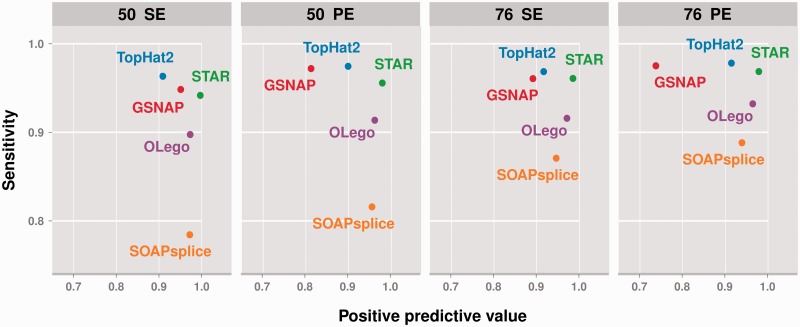
STAR exhibits superior splice junction detection precision, whereas TopHat2 shows the best sensitivity. The plot shows the junction detection sensitivity (y-axis) and positive predictive value (x-axis) of each alignment method, averaged over 10 simulated data sets per experimental condition, at 20 M reads sequencing depth. Each panel corresponds to a different simulation set-up: 50 or 76 bp read length, single-end (SE) or paired-end (PE) library.

The detection of expressed junctions relies on the retrieval of gapped alignment hits (in genome space) from the mapping output. We assessed the sensitivity and positive predictive value of each alignment method by considering as true positives all junctions present in the simulated data spanned by at least one uniquely mapping read, as false positives all gapped alignments which do not correspond to an expressed junction, and as false negatives expressed junctions with no unique hit. The results of the comparison, under all simulation settings, are shown in Figure 2 at 20 M reads sequencing depth and in Supplementary Figure S6 at 8M and 40M. Again, all metrics were averaged over 10 random data sets per experimental condition and low standard deviation was observed across the data sets (Supplementary Table S4). In terms of splice junction detection precision (positive predictive value) STAR exhibits the best performance, with remarkably low amounts of false positive hits and high sensitivity, further improving at increasing read lengths with minor loss in precision. OLego provides as well high precision, though at lower sensitivity, while GSNAP and TopHat2 achieve greater sensitivity but with relatively poor positive predictive value. SOAPsplice does not rely on prior knowledge to detect expressed junctions, yet it proves to achieve a good trade-off between precision and sensitivity, often with noteworthy gains at higher read lengths and with paired-end information.

### Splice junction quantification performance

**Figure 3.**
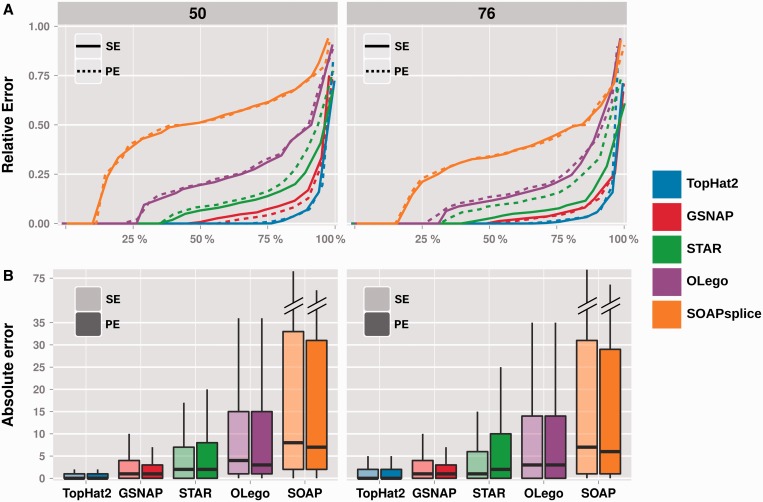
TopHat2 achieves the best quantification accuracy, both in terms of relative and absolute counts of junction-spanning reads. (**A**) The plot shows the relative quantification error (absolute difference between alignment and true counts relative to the true expression value, y-axis) at increasing percentiles (x-axis) for each alignment method and under different simulation set-ups (at 20 M reads sequencing depth), all values being averaged over 10 simulated data sets per experimental condition. Reads of 50 and 76 bp are represented on separate panels, single-end and paired-end reads with, respectively, continuous and dashed lines. (**B**) For each alignment method, the absolute quantification error (absolute difference between alignment and true counts, comprising false-negative and false-positive junctions, y-axis) is shown in a box plot representation encompassing all 10 simulated data sets, at 20 M reads sequencing depth. Reads of 50 and 76 bp are represented on separate panels, single-end (SE) and paired-end (PE) reads with distinct fill gradients.

The quantification of expression levels is carried out by counting the number of uniquely mapping reads spanning a given junction. The absolute difference between true counts and alignment counts has been evaluated over all detectable junctions, i.e. spanned by at least a uniquely mapping read. Additionally, for true-positive ones, the relative error (absolute difference divided by the true expression value) was computed to further discern the impact of quantification errors at different expression ranges. In either case, and consistently with the mapping performance, TopHat2 achieves the best quantification accuracy under all tested experimental conditions. For most true-positive junctions (∼80%, cf. Figure 3A and Supplementary Figure S7) the quantification is exact, with no difference in read counts, and the 1.5 interquartile range of absolute errors across all simulated data sets and experimental settings lies within 5 read counts, with a median of zero. Predictably, the absolute error increases at increasing sequencing depths (see Figure 3B at 20 M reads and Supplementary Figure S8 at 8M and 40M) and read lengths, though with minor discrepancies in relative terms.

### Enhanced splice junction detection and quantification with FineSplice

**Figure 4.**
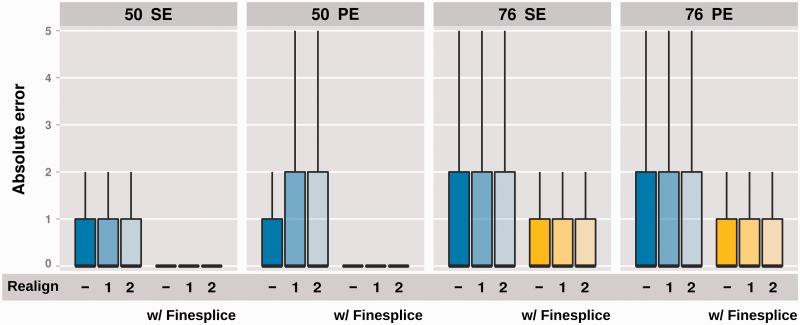
FineSplice achieves better quantification accuracy by filtering out false positive junctions and rescuing multiple mapping reads. TopHat2 absolute quantification error before (blue bars) and after (yellow bars) filtering with FineSplice in a box plot representation (cf. Figure 3B). Different simulation settings at 20 M reads sequencing depth are represented on separate panels: 50 bp or 76 bp read length, single-end (SE) or paired-end (PE) library. Each bar corresponds to different TopHat2 alignment options, either default (−) or with realignment of reads spanning multiple exons, allowing up to 1 or 2 mismatches in read segments alignment.

Our results show that TopHat2 achieves the best results in terms of mapping and quantification precision, but at the undesirable price of a high rate of false detections. To conjugate TopHat2 superior mapping capability with efficient splice junction discovery we propose an integrated pipeline based on a semi-supervised detection of reliable junction alignments. The suggested procedure, FineSplice, has been implemented in the form of a post-processing tool for TopHat2, and evaluated under all simulated conditions (see ‘Materials and Methods’ section). To assess whether false-positive hits could be corrected by more stringent alignment options, we also ran TopHat2 allowing for the realignment of ambiguously mapping multi-exon reads, with different edit distance cut-offs in the initial segment mapping phase. In most cases, TopHat2 with default parameters appears to achieve better results, with only minor improvements in terms of precision (if any) when allowing for the realignment of ambiguously mapping reads (Table 1 and Supplementary Table S5). Our strategy, instead, allows remarkable gain in detection precision, with up to 10% of false-positive junctions being filtered out while having only minor losses in sensitivity. Accordingly, superior F_1_ scores were obtained under all experimental conditions and alignment options (see Table 1 at 20 M reads sequencing depth and Supplementary Table S5 for different library sizes). The effect of filtering false-positive hits and rescuing multiple mapping reads lead, moreover, to a better quantification accuracy both in terms of absolute error (shown in Figure 4 at 20 M reads sequencing depth, and Supplementary Figure S9 at 8 M and 40 M) and relatively to the actual value for true-positive junctions (Supplementary Figure S10).

**Table 1.** FineSplice improves TopHat2 detection precision with small loss in sensitivity and superior F_1_ scores

Read length	Library	Sensitivity		PPV		F_1_ score		Realign w/ segment mismatches
		TopHat2	FineSplice	TopHat2	FineSplice	TopHat2	FineSplice	
**50_bp_**	***SE***	**0.966**	0.941	0.908	**0.989**	0.936	**0.964**	**–**
		**0.966**	0.922	0.919	**0.987**	0.942	**0.953**	**1**
		**0.966**	0.922	0.918	**0.986**	0.941	**0.953**	**2**
	***PE***	**0.978**	0.960	0.906	**0.984**	0.940	**0.972**	**–**
		**0.977**	0.966	0.902	**0.973**	0.938	**0.969**	**1**
		**0.978**	0.966	0.902	**0.973**	0.938	**0.969**	**2**
**76_bp_**	***SE***	**0.967**	0.950	0.919	**0.993**	0.943	**0.971**	**–**
		**0.967**	0.911	0.929	**0.990**	0.947	**0.949**	**1**
		**0.967**	0.911	0.929	**0.990**	0.947	**0.949**	**2**
	***PE***	**0.978**	0.961	0.917	**0.991**	0.947	**0.976**	**–**
		**0.978**	0.971	0.901	**0.974**	0.938	**0.973**	**1**
		**0.978**	0.971	0.901	**0.974**	0.938	**0.973**	**2**

TopHat2 run with default parameters (highlighted) or realignment option (--read-realign-edit-dist 0), allowing up to 1 or 2 mismatches in read segments alignment (--segment-mismatches 1 or 2). SE, single-end; PE, paired-end; PPV, Positive predictive value.

To compare the performance of our pipeline with tools following a somehow similar strategy, we compared FineSplice with TrueSight, a recently released *ab initio* alignment method that uses semi-supervised logistic regression to accurately map junction-spanning reads. Under all simulation settings, the combination of TopHat2 and FineSplice achieves superior detection performance, with higher sensitivity and precision (Supplementary Figure S11). The sensitivity and positive predictive value of inferred junctions were also computed at increasing thresholds for the respective score (posterior probability computed by the two algorithms), with larger dots designating the default behaviour. TrueSight uses model predictions (i.e. the posterior probability) to allocate potentially gapped reads to a reliable mapping location, so by default no threshold is actually applied. Instead, FineSplice uses logistic regression to discriminate potential false-positive junctions from the alignment output, and uses a default threshold of 0.5 to filter out unreliable mapping hits. The score assigned by FineSplice correlates with an improvement in detection precision, and the posterior probability can effectively discriminate false-positive hits (i.e. not expressed junctions) with small loss in sensitivity. On the contrary, TrueSight score does not correlate with a more accurate detection of expressed junctions and thresholding the posterior probability results in a decrease in sensitivity with no apparent improvement in positive predictive value.

### Detection performance in experimental data

We took advantage of publicly available RNA-Seq experiments to evaluate the splice junction detection performance in real data. Three data sets, for eight sequencing runs, were chosen from organisms with variable annotation qualities (human and pig), covering different experimental set-ups (read lengths and library preparation protocols) and per base error rates (low and high per base Phred quality scores, see ‘Materials and Methods’ section). Though in the absence of ground truth it is impossible to make exact statements about the expression status of a splice junction (and hence rigorously define true and false detections), the agreement among different methods and the average number of reads over all alignments can be used as a proxy for evaluating the reliability of a junction hit. We therefore assessed the detection performance by means of pseudo metrics, based on median read counts across alignments, and by unbiasedly evaluating the mean number of reads and concordant detections across alignment algorithms for all splice junctions detected by each method. While not rigorous, these definitions allow for the performance of each method to be evaluated under different assumptions. Pseudo sensitivity and pseudo precision (together with the corresponding F_1_ score) were computed by regarding as true all junctions with a median number of reads across all methods >0. In all three data sets, FineSplice effectively improves TopHat2 performance by filtering a high amount of junctions that were found exclusively by TopHat2 and/or with an average read counts across all alignments as low as 0.25 or 0.5 (Supplementary Figures S12–S14). FineSplice enhances pseudo precision with minor decrease in pseudo sensitivity and achieves superior F_1_ scores in virtually all cases. TrueSight attains high pseudo precision at the expense of a low percentage of detected junctions and poor overall pseudo sensitivity, while SOAPsplice obtains better results and provides a better trade-off for pure *ab initio* discovery. Consistently with the benchmarking results in simulated data, GSNAP usually achieves high pseudo sensitivity at lower precision and detects a high percentage of junctions with a low average read count and consensus over all alignments. However, it performs notably well in the pig data set, achieving superior F_1_ scores though at slightly worse pseudo precision.

To test our assumptions about the expected overhang distribution of reliable versus potential false-positive hits, we further assessed the coverage at each overhang position across all alignment methods for all junctions accepted and rejected by FineSplice. Compared with the set of accepted junctions, the potential false positives discarded by FineSplice show systematically shorter overhangs and low read counts across all alignment methods (Supplementary Figures S15–S17). Consistently with the simulated data, most of the junctions filtered out in all data sets were found exclusively by TopHat2 or by TopHat2 and GSNAP, mainly at low read counts and short overhangs. Overall, even in real data, FineSplice proves to effectively identify unreliable junction hits and achieve greater precision with small loss in sensitivity, providing robust results and the best trade-off in terms of detection performance.

## DISCUSSION

The computational analysis of AS from RNA-Seq data is a complex and rapidly evolving field where, despite the large availability of algorithmic solutions, there is currently a lack of guidelines and best practices are often unclear. Here we intended to provide an effective solution in a common scenario where transcript information is, at least partially, available but novel splicing events still need to be identified *de novo*. We addressed the problem at its most fundamental level: the alignment, detection and quantification of exon–exon junctions from the sequencing data. The objective, in this respect, is to reliably measure and identify the set of splice junctions expressed under a given condition. While ignoring additional layers of interpretation, this intron-centric perspective allows the problem to be dissected to its core and for the evaluation of the impact of design and method in a straightforward manner. An efficient solution to this problem allows to further identify possible sources of bias or confusion in downstream analysis, as any splicing variant must differ by at least one exon junction. Splice junction discovery and quantification itself pose numerous, often opposing, challenges in terms of data analysis and processing, especially when only partial transcript information is available, as is commonly the case.

Here we performed a comprehensive assessment of the performance of different alignment algorithms on simulated data under variable experimental set-ups and annotations sets. Based on the results, we propose a pipeline to fulfil all the desirable prerogatives of an effective expression analysis at the level of splice sites, which, predictably, cannot be attained by any alignment alone. Aside from minor differences related to experimental design, the relative advantage of each approach largely depends on the analysis envisaged because all benchmarked methods exhibit in fact opposing strengths and drawbacks. In our hands, TopHat2 proved to achieve superior results in terms of mapping and quantification accuracy though at the price of producing many spurious gapped alignments, hence introducing more false positives. The greatest advantage of TopHat2 over other aligners is that it makes use of full-length transcripts in its preliminary alignment step. This results in significant gains in sensitivity, mapping accuracy and leads to better quantification results, but at the expense of transcriptome misalignments (particularly with low-quality ends and reads spanning multiple splice sites). On the other hand, false-positive calls are less likely within more stringent mapping schemes and relying on splice site annotations (e.g. OLego and STAR) but, at the same time, this strategy compromises the ability to allocate a considerable amount of reads. Though requiring an efficient downstream strategy to solve the detection problem, relying on a less-conservative alignment method allows to benefit from higher mapping accuracy and better expression estimates. TopHat2 already achieves good standards for mapping and quantification purposes and, albeit penalized by poor false-positive detection rates, it offers a solid basis that can lead to better results through a convenient post-processing of the alignment output.

**Figure 5.**
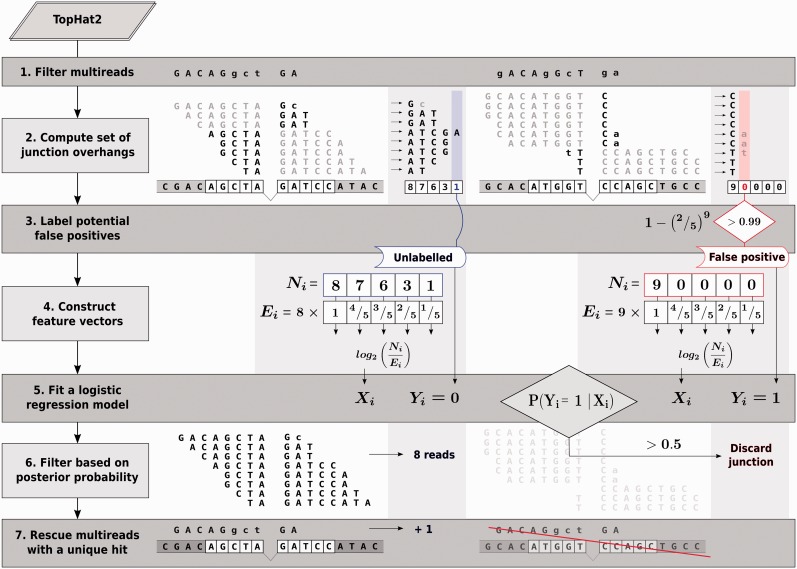
Overview of FineSplice pipeline. Following alignment with TopHat2, non-uniquely mapping reads spanning multiple splice sites are temporarily filtered out (1). The set of split-read overhangs across the junction is then computed (2). A subset of potential false positives is defined (3) based on the probability of observing, at the given read count, at least one overhang greater than the first mismatching position, if none is found. Feature vectors are constructed based on the deviation at each position between observed and expected read counts under uniformity assumptions (4), after trimming mismatching overhangs at the first mismatch position. A logistic regression model is fitted on the labelled subset of potential false positives against the remaining total of detectable junctions (5). After discarding splice junctions with a higher posterior probability of belonging to the false-positive class (6), multireads with a unique location after filtering are assigned to the accepted candidate junction.

We therefore propose an integrated workflow that, starting from TopHat2, aims at optimizing the detection process by filtering out false positives with minimal loss in sensitivity, and rescuing multiple mapping reads whereby a unique location is obtained after filtering. This pipeline allows to take advantage, with further benefits in terms of multiread allocation, of the unequalled quantification accuracy provided by TopHat2, while considerably enhancing precision. This was achieved by developing a post-processing scheme as a Python wrapper for TopHat2, named FineSplice (Figure 5). FineSplice aims at evaluating the reliability of a junction call from TopHat2 alignment and identify a confident set of expressed splice junctions. This is carried out in a semi-supervised fashion by fitting a logistic regression model on the set of aligned reads spanning a given junction against a labelled class of potential false positives.

FineSplice takes as input TopHat2 alignment and outputs a confident set of expressed junctions along with their corresponding counts, through an anomaly detection strategy aiming at the removal of false-positive junctions arising from artefactual or spurious alignments. In our approach, junctions are represented by a set of read overhangs, defined as the shortest segment of a split-read across the splice site. The systematic occurrence of mismatches on the shortest overlapping arm of each read is penalized by trimming all overhangs at the first mismatching position and ignoring subsequent matches. Given the inherent difficulty of defining a category of valid spliced alignments, the main idea is in fact to use the deviation of overhangs from uniformity assumptions to discriminate anomalous junctions affected by systematically shorter overhangs and frequent mismatches. To do this, a subset of potential false positives is first defined. Feature vectors are hence constructed based on the deviation between the observed number of reads spanning a given position and the expected counts, computed in terms of log-ratio. Following logistic regression, posterior probability estimates are used as proxy variables to compare the majority of detectable junction alignments (which are reasonably assumed to be in vast majority proper mappings) against the designated subset of potential false positives. Because counts at distant positions are inherently noisier and not necessarily meaningful to discriminate spurious junctions, a sparse representation of the feature vectors is promoted via L1-regularization on the regression coefficients to select the relevant features in an agnostic way (48).

The initial definition of the subset of false positive examples, though ultimately arbitrary, needs to be strict enough to designate a representative fraction of extremely anomalous mappings as a benchmark for fitting the logistic regression model. Accordingly, a junction is labelled as a potential false positive exclusively when (i) no overhang length on either side of the junction is found to be greater than the first mismatch position and (ii) the probability of observing, at the given number of reads, a longer overhang is greater than a given threshold (here 0.99). Only junctions with a single mismatching position are considered, given that the probability must reflect a patent violation of the uniformity assumptions rather than an ambiguous mapping location. A certain misclassification error is contemplated, as anomalous hits may arise as a consequence of technical and biological confounding factors (e.g. low quality ends, low read counts, polymorphic or repetitive regions) aside from incorrect alignments. However, the chance will be substantially lower if a certain amount of false positives is expected, and minimal compared with the overall amount of detectable junctions. This makes the loss in sensitivity eventually negligible compared with the gain in positive predictive value. After filtering, non-uniquely mapping reads spanning multiple splice sites are rescued whenever a unique candidate location is retrieved after filtering.

Logistic regression is used in both OLego and TrueSight to prioritize candidate mapping locations within the *de novo* splice junction discovery process. An assorted set of features, e.g. sequence consensus and intron size (OLego) plus coding potential and mapping-derived information (TrueSight), is computed for each splice site, and model predictions are used to guide the discovery of novel junctions and allocate initially unmapped reads to a reliable location. This approach allows remarkable detection precision, but at the expense of sensitivity and quantification power, and its advantage is questionable whenever the transcriptome, or part of it, has already been assembled or can be reconstructed *de novo*. In contrast, FineSplice evaluates the reliability of a junction call considering the overall set of split-reads after the alignment is performed. This relaxes the constraints over the mapping process, allowing to place a higher number of reads while tolerating a certain amount of transcriptome misalignments. Constraining the detection problem at the post-processing stage allows better mapping performance while subsequently getting rid of spurious hits, with a minimal loss in the percentage of discarded reads and superior overall quantification power.

In summary, we propose a pipeline to effectively detect and estimate the set of expressed splice junctions from RNA-Seq data, in a typical context where transcript annotations are available but novel isoforms might not be characterized. This demands for both an efficient discovery of novel splice sites and an accurate mapping of known ones, which cannot be easily attained by relying on the sole alignment output. The suggested scheme takes advantage of the better mapping and quantification achievable through transcriptome-first alignment, while later adjusting for inherent biases and filtering unreliable junction hits. To do this, we couple TopHat2 to a novel splice junction detection method, FineSplice, which discards unreliable gapped alignments allowing for an up to 10-fold reduction in the number of false positives and ∼99% precision in detecting expressed features. Both in synthetic data sets with sampled transcript annotations and in real data, FineSplice produces significant gains in precision at small drops in sensitivity. The combination of TopHat2 and FineSplice ultimately provides the best trade-off in terms of mapping, detection and quantification performance, and a simple and effective pipeline for a successful analysis of splicing events at the junction level, that will hopefully ease standard analysis of AS from RNA-Seq data.

## SUPPLEMENTARY DATA

Supplementary Data are available at NAR Online.

## FUNDING

European Union’s FP7 [ERG-239158 to E.L.-P., CardioNeT-ITN-289600 to E.L.-P., S.A.C. and P.J.R.B.]; Spanish Ministry of Science and Innovation [BFU2009-10016, SAF2012-31451 to E.L.-P.]; Regional Government of Madrid [2010-BMD-2321 ‘Fibroteam’ to E.L.-P.]; Spanish Ministry of Economy and Competitiveness and by the Pro-CNIC Foundation (towards CNIC); National Institute for Health Research Cardiovascular BRU at the Royal Brompton & Harefield NHS Foundation Trust and Imperial College London (to S.A.C. and P.J.B.). Funding for open access charge: European Union’s FP7 [ERG-239158, CardioNeT-ITN-289600 to E.L.-P.].

*Conflict of interest statement*. None declared.

## Supplementary Material

Supplementary Data
