# Supplementary material for: FineSplice, enhanced splice junction detection and quantification: a novel pipeline based on the assessment of diverse RNA-Seq alignment solutions
Source: Nucleic Acids Res. 2014 Feb 25;42(8):e71. doi: 10.1093/nar/gku166 (PMC4005686; doi:10.1093/nar/gku166)
Supplement: Supplementary Data [file supp_42_8_e71__index.html]

FineSplice, enhanced splice junction detection and quantification: a novel pipeline based on the assessment of diverse RNA-Seq alignment solutions — Supplementary Data 

# FineSplice, enhanced splice junction detection and quantification: a novel pipeline based on the assessment of diverse RNA-Seq alignment solutions

## Supplementary Data

files

**Files in this Data Supplement:**

- Supplementary Data - pdf file
