# Supplementary material for: FineSplice, enhanced splice junction detection and quantification: a novel pipeline based on the assessment of diverse RNA-Seq alignment solutions
Source: Nucleic Acids Res. 2014 Feb 25;42(8):e71. doi: 10.1093/nar/gku166 (PMC4005686; doi:10.1093/nar/gku166)
Supplement: Supplementary Data [file supp_gku166_nar-03284-met-n-2013-File008.pdf]

Supplementary Table 1. Alignment precision computed over all uniquely mapped reads

Reads	Read length	Type	Metric	GSNAP		TopHat2		STAR		OLego		SOAPsplice	
8M	50_{bp}	SE	PPV	0.993	± 0.000	0.992	± 0.001	0.985	± 0.000	0.845	± 0.004	0.788	± 0.005
			Unique (%)	79.633	± 0.609	80.776	± 0.545	80.447	± 0.604	90.665	± 0.361	95.226	± 0.101
			Unmapped (%)	15.533	± 0.299	15.167	± 0.291	14.595	± 0.318	4.422	± 0.093	4.588	± 0.099
		PE	PPV	0.988	± 0.000	0.992	± 0.001	0.984	± 0.001	0.846	± 0.004	0.928	± 0.002
			Unique (%)	82.732	± 0.668	81.723	± 0.608	77.875	± 0.747	90.773	± 0.509	74.886	± 0.656
			Unmapped (%)	14.124	± 0.411	15.072	± 0.435	19.204	± 0.563	4.398	± 0.095	24.912	± 0.640
	76_{bp}	SE	PPV	0.974	± 0.001	0.995	± 0.001	0.981	± 0.001	0.862	± 0.003	0.806	± 0.004
			Unique (%)	83.453	± 0.507	80.342	± 0.506	81.703	± 0.549	87.974	± 0.452	92.409	± 0.148
			Unmapped (%)	12.599	± 0.247	16.523	± 0.294	14.612	± 0.285	6.667	± 0.105	7.165	± 0.123
		PE	PPV	0.974	± 0.001	0.995	± 0.000	0.977	± 0.000	0.862	± 0.002	0.848	± 0.002
			Unique (%)	84.802	± 0.488	81.078	± 0.456	79.378	± 0.473	87.766	± 0.515	84.188	± 0.466
			Unmapped (%)	12.432	± 0.175	16.461	± 0.219	18.079	± 0.278	6.655	± 0.084	15.378	± 0.415
20M	50_{bp}	SE	PPV	0.993	± 0.000	0.992	± 0.001	0.985	± 0.000	0.845	± 0.005	0.788	± 0.004
			Unique (%)	79.588	± 0.478	80.715	± 0.434	80.382	± 0.459	90.585	± 0.171	95.243	± 0.073
			Unmapped (%)	15.563	± 0.489	15.194	± 0.483	14.611	± 0.514	4.401	± 0.101	4.574	± 0.073
		PE	PPV	0.988	± 0.000	0.991	± 0.005	0.985	± 0.000	0.848	± 0.003	0.929	± 0.002
			Unique (%)	82.969	± 0.543	82.028	± 0.447	78.199	± 0.557	90.822	± 0.544	75.068	± 0.537
			Unmapped (%)	13.782	± 0.297	14.721	± 0.317	18.853	± 0.434	4.328	± 0.076	24.716	± 0.532
	76_{bp}	SE	PPV	0.974	± 0.001	0.995	± 0.000	0.981	± 0.001	0.863	± 0.004	0.808	± 0.004
			Unique (%)	83.476	± 0.473	80.404	± 0.555	81.739	± 0.506	87.926	± 0.404	92.361	± 0.226
			Unmapped (%)	12.541	± 0.307	16.444	± 0.371	14.553	± 0.374	6.65	± 0.128	7.174	± 0.121
		PE	PPV	0.974	± 0.001	0.995	± 0.001	0.977	± 0.000	0.86	± 0.005	0.848	± 0.003
			Unique (%)	84.731	± 0.296	80.888	± 0.337	79.212	± 0.425	88.404	± 1.679	84.337	± 0.356
			Unmapped (%)	12.546	± 0.338	16.612	± 0.440	18.272	± 0.494	6.692	± 0.161	15.233	± 0.376
40M	50_{bp}	SE	PPV	0.993	± 0.000	0.993	± 0.001	0.985	± 0.000	0.843	± 0.004	0.786	± 0.005
			Unique (%)	79.381	± 0.532	80.56	± 0.556	80.19	± 0.519	90.482	± 0.414	95.221	± 0.067
			Unmapped (%)	15.7	± 0.413	15.319	± 0.403	14.721	± 0.408	4.447	± 0.081	4.596	± 0.061
		PE	PPV	0.988	± 0.000	0.992	± 0.000	0.984	± 0.000	0.846	± 0.004	0.928	± 0.002
			Unique (%)	82.916	± 0.571	81.878	± 0.541	78.027	± 0.620	90.979	± 0.277	74.944	± 0.631
			Unmapped (%)	14.12	± 0.330	15.074	± 0.341	19.21	± 0.439	4.406	± 0.058	24.847	± 0.608
	76_{bp}	SE	PPV	0.973	± 0.001	0.995	± 0.000	0.98	± 0.001	0.861	± 0.003	0.805	± 0.004
			Unique (%)	83.227	± 0.595	80.088	± 0.645	81.464	± 0.603	87.769	± 0.487	92.402	± 0.111
			Unmapped (%)	12.685	± 0.206	16.637	± 0.263	14.716	± 0.249	6.712	± 0.099	7.175	± 0.072
		PE	PPV	0.974	± 0.001	0.994	± 0.001	0.976	± 0.001	0.861	± 0.004	0.848	± 0.004
			Unique (%)	84.73	± 0.513	80.973	± 0.575	79.229	± 0.643	87.643	± 0.323	84.171	± 0.406
			Unmapped (%)	12.502	± 0.353	16.567	± 0.462	18.199	± 0.552	6.679	± 0.156	15.39	± 0.395

Supplementary Table 2. Alignment precision computed over all uniquely mapped reads spanning known junctions

				GSNAP		TopHat2		STAR		OLego	
8M	50bp	SE	PPV	0.974	± 0.000	0.999	± 0.000	0.954	± 0.001	0.908	± 0.002
			Unique (%)	94.425	± 0.294	94.795	± 0.343	93.496	± 0.335	84.319	± 0.387
			Unmapped (%)	1.121	± 0.097	0.724	± 0.051	0.277	± 0.032	5.815	± 0.236
		PE	PPV	0.98	± 0.001	0.998	± 0.000	0.951	± 0.000	0.906	± 0.001
			Unique (%)	96.769	± 0.376	95.504	± 0.308	92.088	± 0.367	85.447	± 0.361
			Unmapped (%)	0.49	± 0.090	0.775	± 0.118	4.972	± 0.262	5.777	± 0.156
	76bp	SE	PPV	0.974	± 0.001	0.999	± 0.000	0.967	± 0.001	0.94	± 0.001
			Unique (%)	95.995	± 0.388	94.761	± 0.320	95.776	± 0.355	85.688	± 0.630
			Unmapped (%)	0.088	± 0.016	1.587	± 0.156	0.48	± 0.083	3.288	± 0.141
		PE	PPV	0.976	± 0.001	0.998	± 0.000	0.95	± 0.001	0.94	± 0.001
			Unique (%)	97.273	± 0.278	95.453	± 0.258	93.324	± 0.262	86.539	± 0.558
			Unmapped (%)	0.065	± 0.015	1.566	± 0.081	3.914	± 0.174	3.302	± 0.123
20M	50bp	SE	PPV	0.974	± 0.000	0.999	± 0.000	0.955	± 0.001	0.908	± 0.003
			Unique (%)	94.454	± 0.300	94.891	± 0.280	93.467	± 0.357	84.285	± 0.530
			Unmapped (%)	1.177	± 0.083	0.764	± 0.065	0.318	± 0.076	5.733	± 0.114
		PE	PPV	0.98	± 0.000	0.998	± 0.000	0.951	± 0.000	0.907	± 0.001
			Unique (%)	96.628	± 0.357	95.301	± 0.334	92.141	± 0.436	85.202	± 0.400
			Unmapped (%)	0.4	± 0.039	0.699	± 0.055	4.806	± 0.298	5.772	± 0.118
	76bp	SE	PPV	0.974	± 0.001	0.998	± 0.000	0.968	± 0.001	0.941	± 0.001
			Unique (%)	95.774	± 0.919	94.413	± 0.899	95.463	± 0.928	85.535	± 1.064
			Unmapped (%)	0.125	± 0.081	1.687	± 0.188	0.598	± 0.173	3.445	± 0.164
		PE	PPV	0.976	± 0.001	0.998	± 0.000	0.95	± 0.000	0.938	± 0.007
			Unique (%)	97.109	± 0.445	95.214	± 0.410	93.079	± 0.450	87.679	± 3.101
			Unmapped (%)	0.077	± 0.020	1.595	± 0.115	3.936	± 0.184	3.293	± 0.118
40M	50bp	SE	PPV	0.974	± 0.000	0.999	± 0.000	0.955	± 0.001	0.908	± 0.001
			Unique (%)	94.307	± 0.335	94.903	± 0.357	93.375	± 0.295	84.111	± 0.575
			Unmapped (%)	1.189	± 0.137	0.732	± 0.095	0.325	± 0.098	5.815	± 0.097
		PE	PPV	0.98	± 0.000	0.998	± 0.000	0.951	± 0.001	0.905	± 0.004
			Unique (%)	96.454	± 0.908	95.208	± 0.818	91.853	± 0.917	85.069	± 0.667
			Unmapped (%)	0.434	± 0.031	0.734	± 0.041	4.876	± 0.286	5.82	± 0.111
	76bp	SE	PPV	0.974	± 0.001	0.998	± 0.000	0.967	± 0.001	0.942	± 0.001
			Unique (%)	96.005	± 0.651	94.571	± 0.558	95.706	± 0.633	85.629	± 0.902
			Unmapped (%)	0.095	± 0.028	1.63	± 0.093	0.524	± 0.078	3.375	± 0.087
		PE	PPV	0.975	± 0.001	0.998	± 0.000	0.95	± 0.001	0.94	± 0.001
			Unique (%)	97.216	± 0.436	95.229	± 0.425	93.195	± 0.506	86.25	± 0.555
			Unmapped (%)	0.089	± 0.041	1.67	± 0.155	3.91	± 0.253	3.366	± 0.185

Supplementary Table 3. Alignment precision computed over all uniquely mapped reads spanning novel junctions

Reads	Length	Type	Metric	GSNAP		TopHat2		STAR		OLego		SOAPsplice	
8M	50_{bp}	SE	PPV	0.892	± 0.010	0.955	± 0.012	0.779	± 0.011	0.885	± 0.007	0.709	± 0.003
			Unique (%)	57.148	± 4.497	81.237	± 5.222	90.915	± 1.833	66.846	± 5.322	81.498	± 0.308
			Unmapped (%)	39.407	± 4.140	11.86	± 5.264	0.979	± 0.725	24.786	± 5.312	17.436	± 0.253
		PE	PPV	0.851	± 0.026	0.942	± 0.053	0.769	± 0.031	0.886	± 0.014	0.762	± 0.002
			Unique (%)	91.111	± 1.856	85.545	± 2.632	88.082	± 2.239	68.43	± 3.945	76.631	± 0.266
			Unmapped (%)	5.382	± 0.969	8.411	± 1.859	8.071	± 1.649	22.986	± 2.733	22.243	± 0.190
	76_{bp}	SE	PPV	0.88	± 0.015	0.952	± 0.016	0.839	± 0.016	0.929	± 0.007	0.815	± 0.005
			Unique (%)	95.079	± 1.709	82.698	± 4.804	93.725	± 3.222	72.628	± 4.936	85.755	± 0.319
			Unmapped (%)	0.208	± 0.300	12.953	± 4.484	1.161	± 0.993	18.496	± 3.857	12.646	± 0.262
		PE	PPV	0.882	± 0.020	0.97	± 0.008	0.84	± 0.011	0.93	± 0.004	0.841	± 0.002
			Unique (%)	96.645	± 1.722	83.056	± 3.900	90.177	± 4.212	74.37	± 2.844	83.917	± 0.387
			Unmapped (%)	0.023	± 0.019	12.453	± 2.431	5.973	± 2.560	17.859	± 1.973	14.477	± 0.225
20M	50_{bp}	SE	PPV	0.874	± 0.041	0.948	± 0.015	0.759	± 0.033	0.881	± 0.011	0.708	± 0.006
			Unique (%)	56.927	± 2.271	82.368	± 4.640	91.044	± 2.726	68.583	± 4.685	81.604	± 0.175
			Unmapped (%)	38.182	± 2.672	8.973	± 1.783	0.827	± 0.608	21.561	± 2.151	17.347	± 0.144
		PE	PPV	0.85	± 0.014	0.94	± 0.032	0.776	± 0.011	0.886	± 0.011	0.763	± 0.002
			Unique (%)	90.198	± 2.474	84.705	± 3.947	88.285	± 2.559	70.165	± 3.292	76.572	± 0.383
			Unmapped (%)	6.207	± 1.295	9.101	± 2.281	8.254	± 1.885	23.08	± 2.750	22.225	± 0.282
	76_{bp}	SE	PPV	0.892	± 0.007	0.963	± 0.010	0.851	± 0.008	0.929	± 0.004	0.816	± 0.004
			Unique (%)	94.786	± 2.508	82.333	± 2.764	93.445	± 2.607	73.506	± 1.949	85.489	± 0.753
			Unmapped (%)	0.162	± 0.120	12.545	± 2.595	0.647	± 0.229	17.825	± 2.173	12.771	± 0.296
		PE	PPV	0.88	± 0.014	0.957	± 0.015	0.829	± 0.009	0.919	± 0.022	0.844	± 0.002
			Unique (%)	96.874	± 1.669	84.125	± 3.757	91.53	± 2.139	75.385	± 3.111	84.106	± 0.323
			Unmapped (%)	0.039	± 0.041	12.01	± 3.939	5.464	± 1.453	17.469	± 3.102	14.296	± 0.190
40M	50_{bp}	SE	PPV	0.896	± 0.012	0.964	± 0.009	0.78	± 0.016	0.889	± 0.008	0.707	± 0.005
			Unique (%)	57.425	± 3.548	84.291	± 3.224	91.6	± 2.276	69.378	± 4.214	81.582	± 0.218
			Unmapped (%)	38.395	± 4.281	8.204	± 1.888	0.709	± 0.340	22.495	± 3.795	17.373	± 0.172
		PE	PPV	0.851	± 0.014	0.936	± 0.029	0.775	± 0.011	0.884	± 0.010	0.76	± 0.003
			Unique (%)	91.965	± 1.302	85.946	± 2.551	89.424	± 2.518	70.052	± 2.104	76.391	± 0.707
			Unmapped (%)	5.479	± 1.276	8.638	± 3.036	7.123	± 1.932	22.773	± 2.821	22.441	± 0.552
	76_{bp}	SE	PPV	0.894	± 0.015	0.962	± 0.016	0.85	± 0.013	0.931	± 0.007	0.816	± 0.005
			Unique (%)	95.806	± 1.935	83.378	± 3.146	94.702	± 2.198	75.558	± 3.717	85.746	± 0.246
			Unmapped (%)	0.072	± 0.073	11.958	± 2.522	0.521	± 0.234	15.717	± 1.739	12.663	± 0.125
		PE	PPV	0.872	± 0.017	0.943	± 0.028	0.833	± 0.013	0.924	± 0.006	0.843	± 0.002
			Unique (%)	96.792	± 1.688	83.07	± 3.084	91.606	± 2.653	71.622	± 4.171	83.972	± 0.280
			Unmapped (%)	0.015	± 0.009	12.836	± 2.883	4.687	± 0.946	17.885	± 1.924	14.404	± 0.145

Supplementary Table 4. Splice junction detection performance of all alignment methods

				GSNAP		TopHat2		STAR		OLego		SOAPsplice	
8M	50bp	SE	PPV	0.96	± 0.001	0.919	± 0.002	0.997	± 0.000	0.979	± 0.001	0.976	± 0.001
			Sensitivity	0.946	± 0.001	0.963	± 0.001	0.939	± 0.002	0.89	± 0.002	0.767	± 0.002
			F_1 score	0.953	± 0.001	0.94	± 0.001	0.967	± 0.001	0.932	± 0.001	0.859	± 0.002
		PE	PPV	0.862	± 0.002	0.917	± 0.001	0.986	± 0.001	0.973	± 0.001	0.967	± 0.001
			Sensitivity	0.971	± 0.002	0.974	± 0.001	0.953	± 0.002	0.906	± 0.002	0.797	± 0.003
			F_1 score	0.913	± 0.001	0.945	± 0.001	0.969	± 0.001	0.938	± 0.001	0.874	± 0.002
	76bp	SE	PPV	0.917	± 0.002	0.93	± 0.002	0.99	± 0.001	0.98	± 0.000	0.958	± 0.002
			Sensitivity	0.96	± 0.001	0.969	± 0.001	0.96	± 0.001	0.91	± 0.001	0.86	± 0.001
			F_1 score	0.938	± 0.001	0.949	± 0.001	0.975	± 0.001	0.944	± 0.001	0.906	± 0.001
		PE	PPV	0.806	± 0.002	0.929	± 0.001	0.984	± 0.001	0.975	± 0.001	0.952	± 0.002
			Sensitivity	0.975	± 0.001	0.977	± 0.001	0.966	± 0.001	0.927	± 0.002	0.878	± 0.002
			F_1 score	0.882	± 0.001	0.953	± 0.001	0.975	± 0.001	0.95	± 0.001	0.913	± 0.001
20M	50bp	SE	PPV	0.951	± 0.001	0.908	± 0.002	0.997	± 0.000	0.973	± 0.001	0.972	± 0.001
			Sensitivity	0.949	± 0.002	0.965	± 0.001	0.943	± 0.002	0.9	± 0.002	0.786	± 0.003
			F_1 score	0.95	± 0.001	0.936	± 0.001	0.969	± 0.001	0.935	± 0.001	0.869	± 0.002
		PE	PPV	0.814	± 0.003	0.903	± 0.002	0.983	± 0.001	0.965	± 0.001	0.959	± 0.002
			Sensitivity	0.973	± 0.001	0.976	± 0.001	0.958	± 0.001	0.915	± 0.002	0.817	± 0.003
			F_1 score	0.887	± 0.002	0.938	± 0.001	0.97	± 0.001	0.94	± 0.001	0.882	± 0.002
	76bp	SE	PPV	0.894	± 0.001	0.92	± 0.001	0.988	± 0.001	0.974	± 0.001	0.949	± 0.003
			Sensitivity	0.962	± 0.001	0.97	± 0.001	0.962	± 0.002	0.918	± 0.002	0.872	± 0.002
			F_1 score	0.927	± 0.001	0.944	± 0.001	0.975	± 0.001	0.945	± 0.001	0.909	± 0.002
		PE	PPV	0.741	± 0.003	0.917	± 0.002	0.981	± 0.001	0.968	± 0.001	0.942	± 0.002
			Sensitivity	0.977	± 0.001	0.979	± 0.001	0.97	± 0.001	0.934	± 0.002	0.89	± 0.002
			F_1 score	0.843	± 0.002	0.947	± 0.001	0.976	± 0.001	0.95	± 0.001	0.915	± 0.001
40M	50bp	SE	PPV	0.946	± 0.001	0.903	± 0.001	0.996	± 0.000	0.968	± 0.001	0.97	± 0.001
			Sensitivity	0.95	± 0.001	0.965	± 0.001	0.944	± 0.001	0.905	± 0.001	0.795	± 0.002
			F_1 score	0.948	± 0.000	0.933	± 0.001	0.97	± 0.000	0.936	± 0.001	0.874	± 0.001
		PE	PPV	0.78	± 0.003	0.896	± 0.001	0.982	± 0.001	0.959	± 0.001	0.955	± 0.001
			Sensitivity	0.974	± 0.001	0.977	± 0.001	0.96	± 0.001	0.92	± 0.001	0.825	± 0.003
			F_1 score	0.866	± 0.002	0.935	± 0.001	0.971	± 0.001	0.939	± 0.001	0.885	± 0.002
	76bp	SE	PPV	0.879	± 0.002	0.915	± 0.001	0.987	± 0.000	0.969	± 0.001	0.942	± 0.002
			Sensitivity	0.961	± 0.001	0.969	± 0.001	0.962	± 0.001	0.922	± 0.001	0.878	± 0.002
			F_1 score	0.919	± 0.001	0.941	± 0.001	0.974	± 0.001	0.945	± 0.001	0.909	± 0.001
		PE	PPV	0.693	± 0.004	0.91	± 0.002	0.979	± 0.001	0.961	± 0.001	0.933	± 0.003
			Sensitivity	0.978	± 0.001	0.98	± 0.001	0.971	± 0.001	0.937	± 0.001	0.895	± 0.002
			F_1 score	0.811	± 0.003	0.944	± 0.001	0.975	± 0.001	0.949	± 0.001	0.914	± 0.001

Supplementary Table 5. Splice junction detection performance of TopHat2 with and without FineSplice

			Sensitivity		PPV		F_1 score		
			TopHat2	FineSplice	TopHat2	FineSplice	TopHat2	FineSplice	Realign w/ segment mismatches
8M	50_{bp}	SE	**0.963**	0.939	0.920	**0.982**	0.941	**0.960**	–
			**0.963**	0.930	0.929	**0.977**	0.946	**0.953**	1
			**0.963**	0.922	0.929	**0.977**	0.946	**0.948**	2
		PE	**0.973**	0.954	0.919	**0.985**	0.945	**0.969**	–
			**0.972**	0.955	0.921	**0.983**	0.946	**0.969**	1
			**0.972**	0.955	0.921	**0.983**	0.946	**0.969**	2
	76_{bp}	SE	**0.968**	0.943	0.930	**0.994**	0.949	**0.968**	–
			**0.967**	0.927	0.939	**0.995**	0.953	**0.960**	1
			**0.967**	0.927	0.939	**0.995**	0.953	**0.960**	2
		PE	**0.977**	0.965	0.929	**0.989**	0.952	**0.977**	–
			**0.976**	0.966	0.925	**0.987**	0.950	**0.976**	1
			**0.976**	0.966	0.925	**0.987**	0.950	**0.976**	2
20M	50_{bp}	SE	**0.966**	0.941	0.908	**0.989**	0.936	**0.964**	–
			**0.966**	0.922	0.919	**0.987**	0.942	**0.953**	1
			**0.966**	0.922	0.918	**0.986**	0.941	**0.953**	2
		PE	**0.978**	0.960	0.906	**0.984**	0.940	**0.972**	–
			**0.977**	0.966	0.902	**0.973**	0.938	**0.969**	1
			**0.978**	0.966	0.902	**0.973**	0.938	**0.969**	2
	76_{bp}	SE	**0.967**	0.950	0.919	**0.993**	0.943	**0.971**	–
			**0.967**	0.911	0.929	**0.990**	0.947	**0.949**	1
			**0.967**	0.911	0.929	**0.990**	0.947	**0.949**	2
		PE	**0.978**	0.961	0.917	**0.991**	0.947	**0.976**	–
			**0.978**	0.971	0.901	**0.974**	0.938	**0.973**	1
			**0.978**	0.971	0.901	**0.974**	0.938	**0.973**	2
40M	50_{bp}	SE	**0.966**	0.941	0.905	**0.989**	0.934	**0.964**	–
			**0.966**	0.922	0.916	**0.991**	0.940	**0.955**	1
			**0.966**	0.925	0.915	**0.991**	0.940	**0.956**	2
		PE	**0.977**	0.955	0.897	**0.984**	0.936	**0.970**	–
			**0.977**	0.960	0.892	**0.977**	0.933	**0.968**	1
			**0.977**	0.960	0.892	**0.977**	0.932	**0.968**	2
	76_{bp}	SE	**0.969**	0.943	0.916	**0.994**	0.942	**0.968**	–
			**0.969**	0.935	0.925	**0.994**	0.947	**0.963**	1
			**0.969**	0.935	0.925	**0.994**	0.947	**0.963**	2
		PE	**0.981**	0.965	0.913	**0.990**	0.946	**0.977**	–
			**0.981**	0.970	0.887	**0.982**	0.932	**0.976**	1
			**0.981**	0.970	0.887	**0.982**	0.932	**0.976**	2

Supplementary Figure 1. Summary table and quality plots for the experimental data used for estimating the 50 bp error profile (SRA Experiment SRX424347, run accession number on top panel)

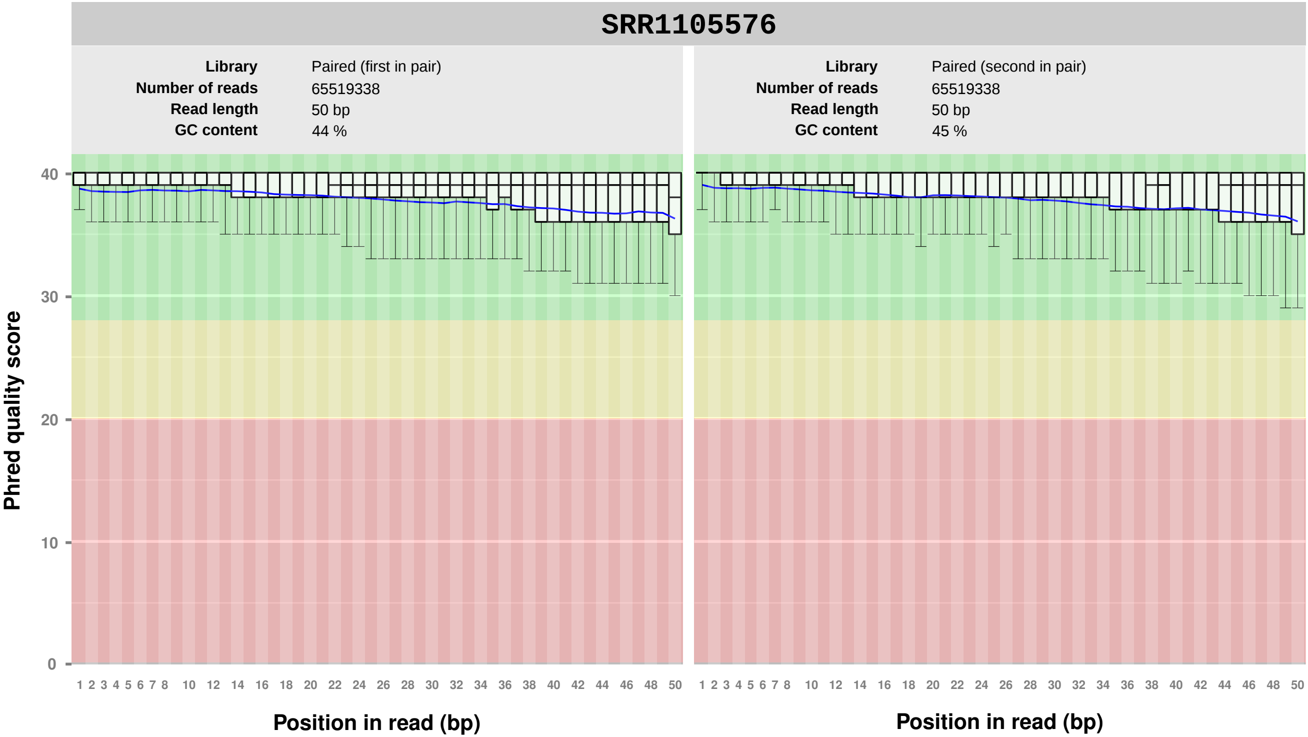

Supplementary Figure 2. Summary tables and quality plots for the human high-quality dataset (SRA Experiment SRX084679, run accession number on top panel)

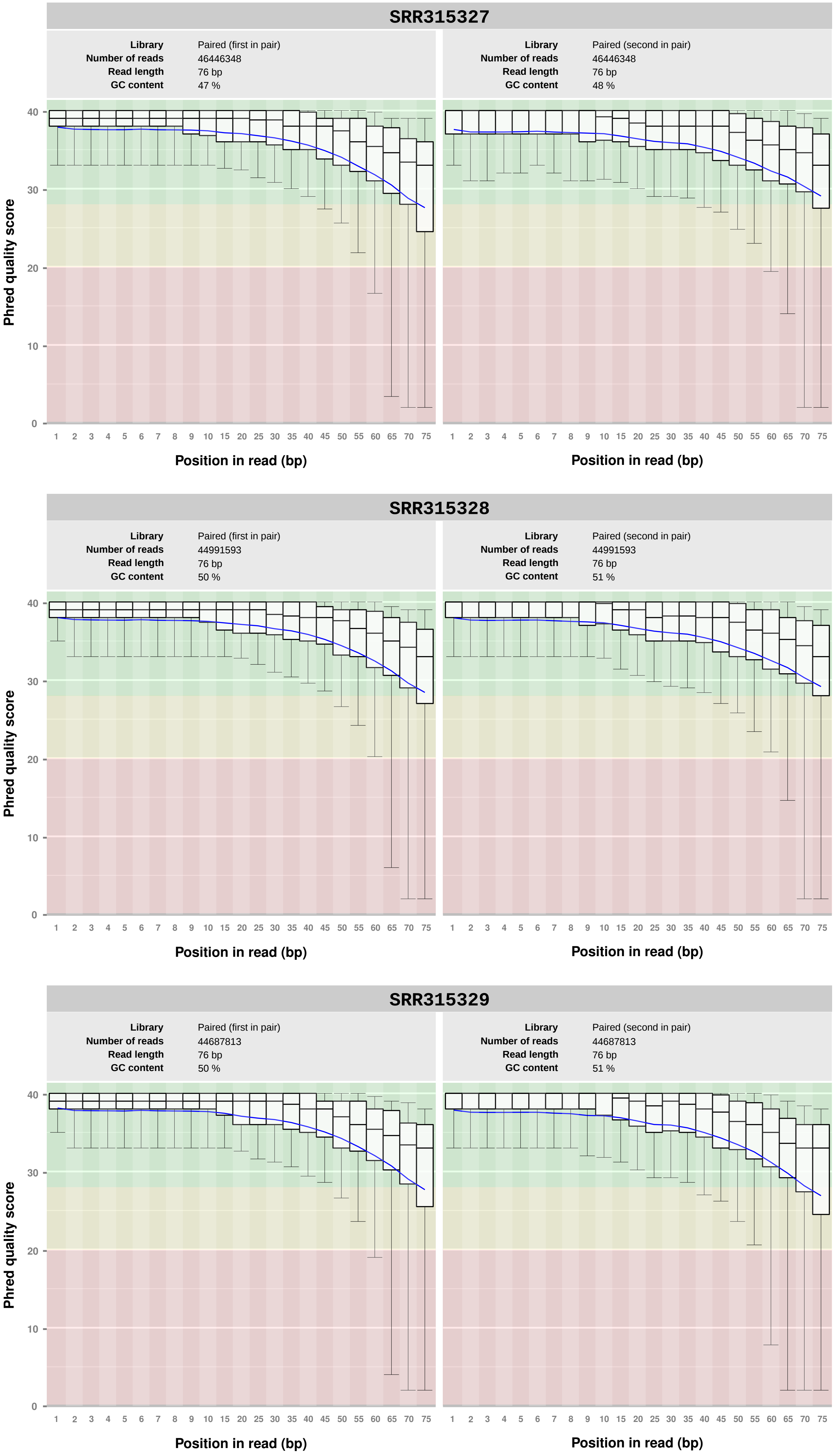

Supplementary Figure 3. Summary tables and quality plots for the human low-quality dataset (SRA Experiment SRX011546, run accession number on top panel)

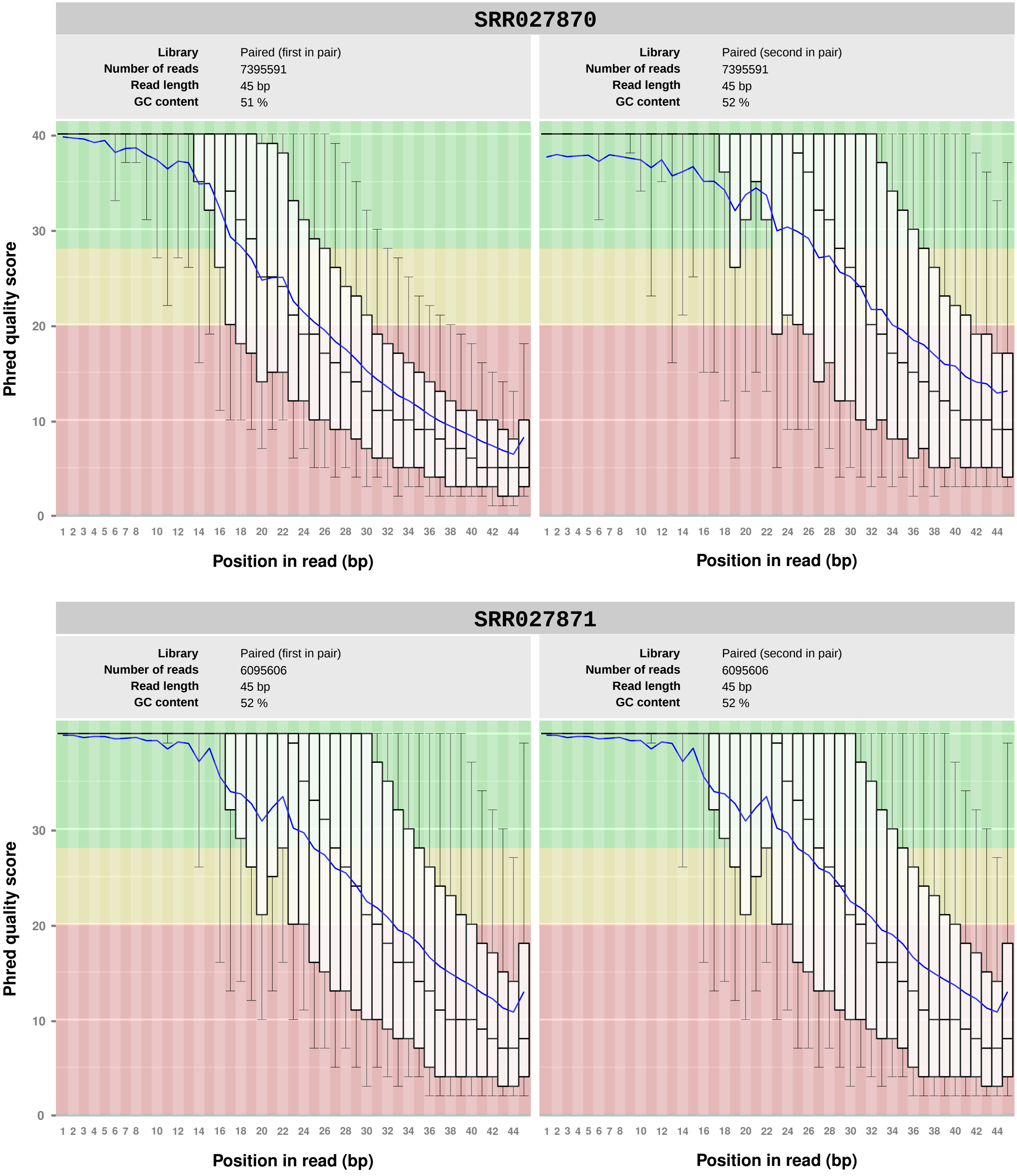

Supplementary Figure 4. Summary tables and quality plots for the pig dataset (SRA Experiments SRX242929, SRX242930 and SRX242931, run accession number on top panel)

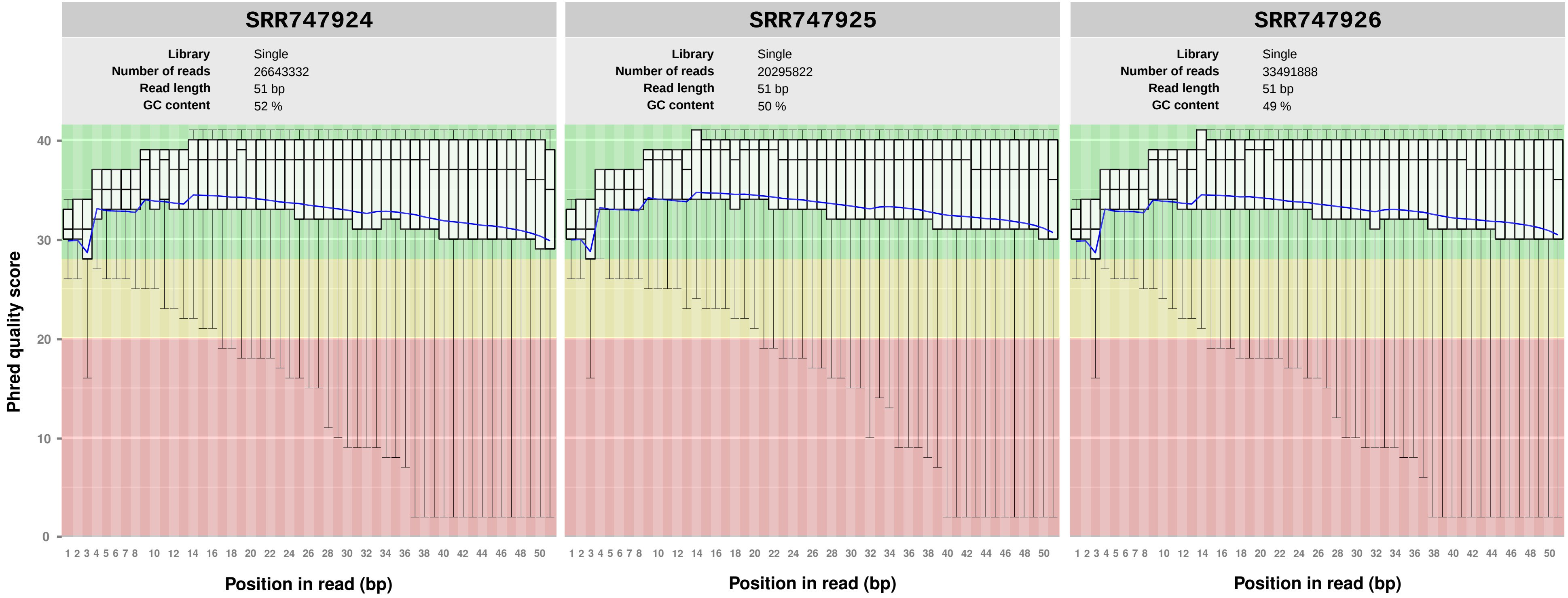

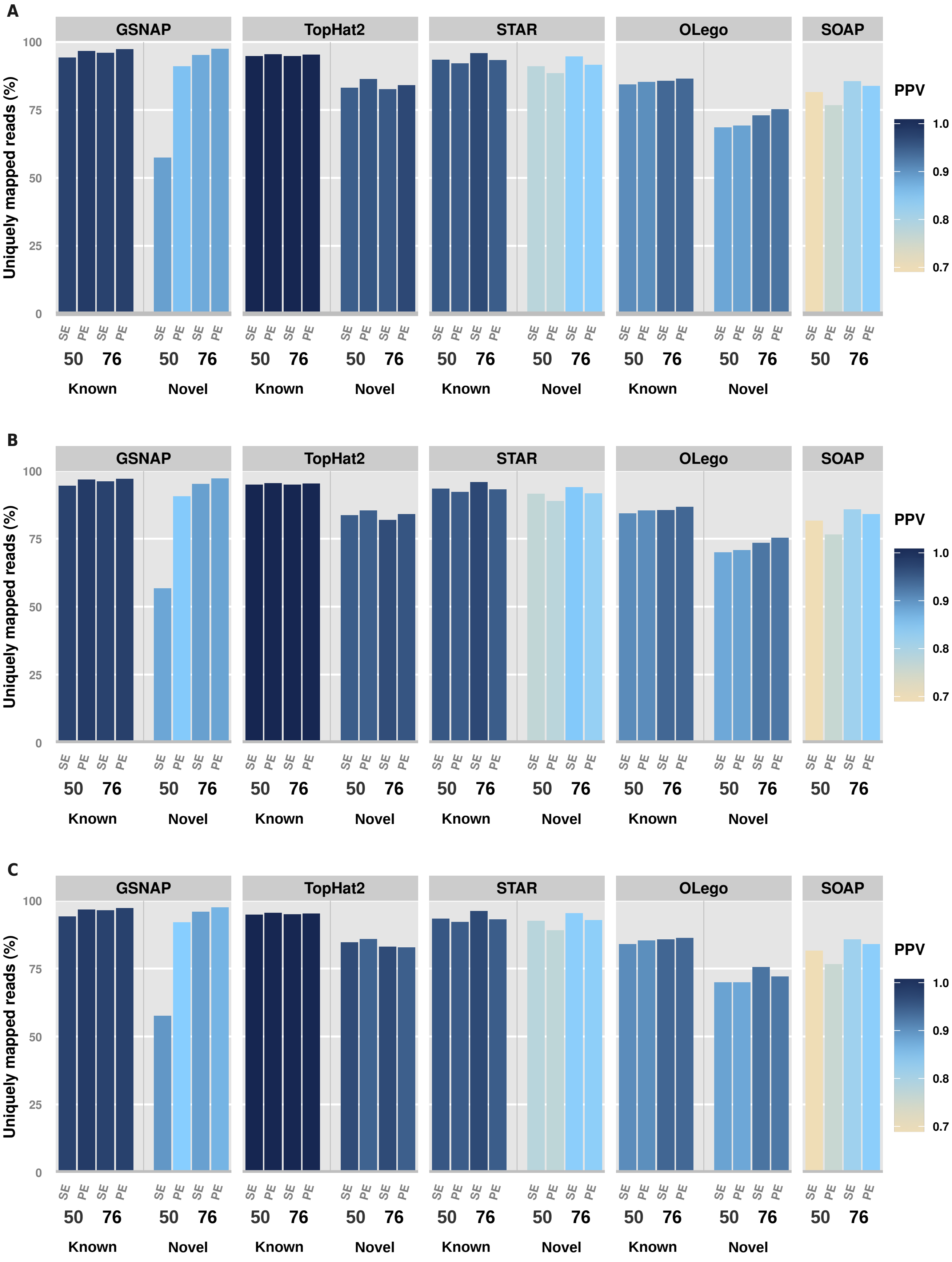

Supplementary Figure 5. Percentage of uniquely mapped reads (bar chart, *y*-axis) and positive predictive value (ratio of correctly aligned nucleotides, color coded), averaged over 10 simulated data sets per experimental condition, for each alignment method (on a separate panel). Left and right sides of each panel correspond to reads spanning known and novel junctions (except for SOAPsplice, which aligns exclusively *ab initio*) Each bar corresponds to a different simulation setup: 50 or 76 bp read length, single-end (SE) or paired-end (PE) library, at 8M (**A**), 20M (**B**) and 40M (**C**) sequencing depth.

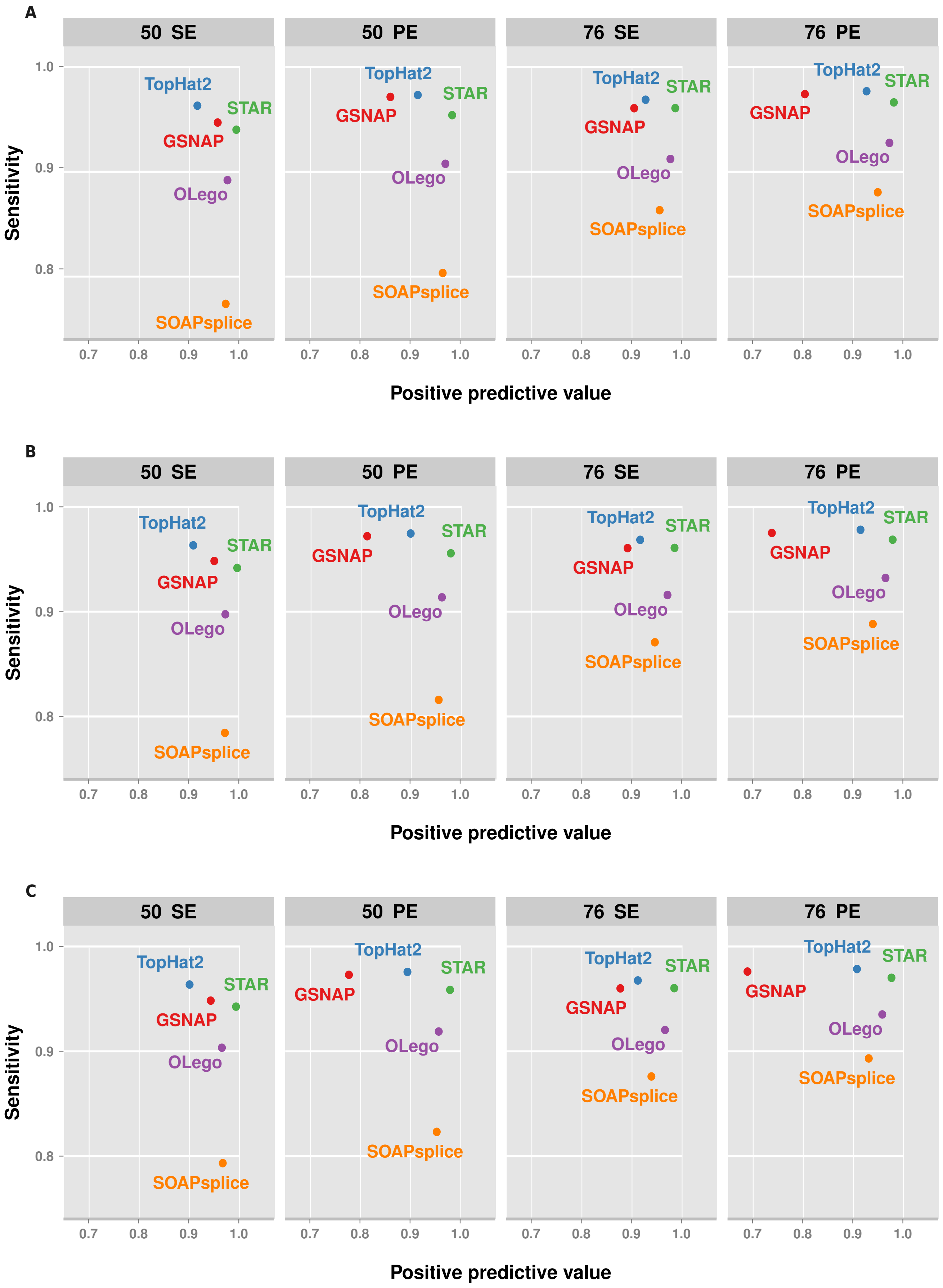

Supplementary Figure 6. Junction detection sensitivity (*y*-axis) and positive predictive value (*x*-axis), averaged over 10 simulated data sets per experimental condition, at 8M (**A**), 20M (**B**) and 40M (**C**) reads sequencing depth. Panels correspond to different simulation setups: 50 or 76 bp read length, single-end (SE) or paired-end (PE) library.

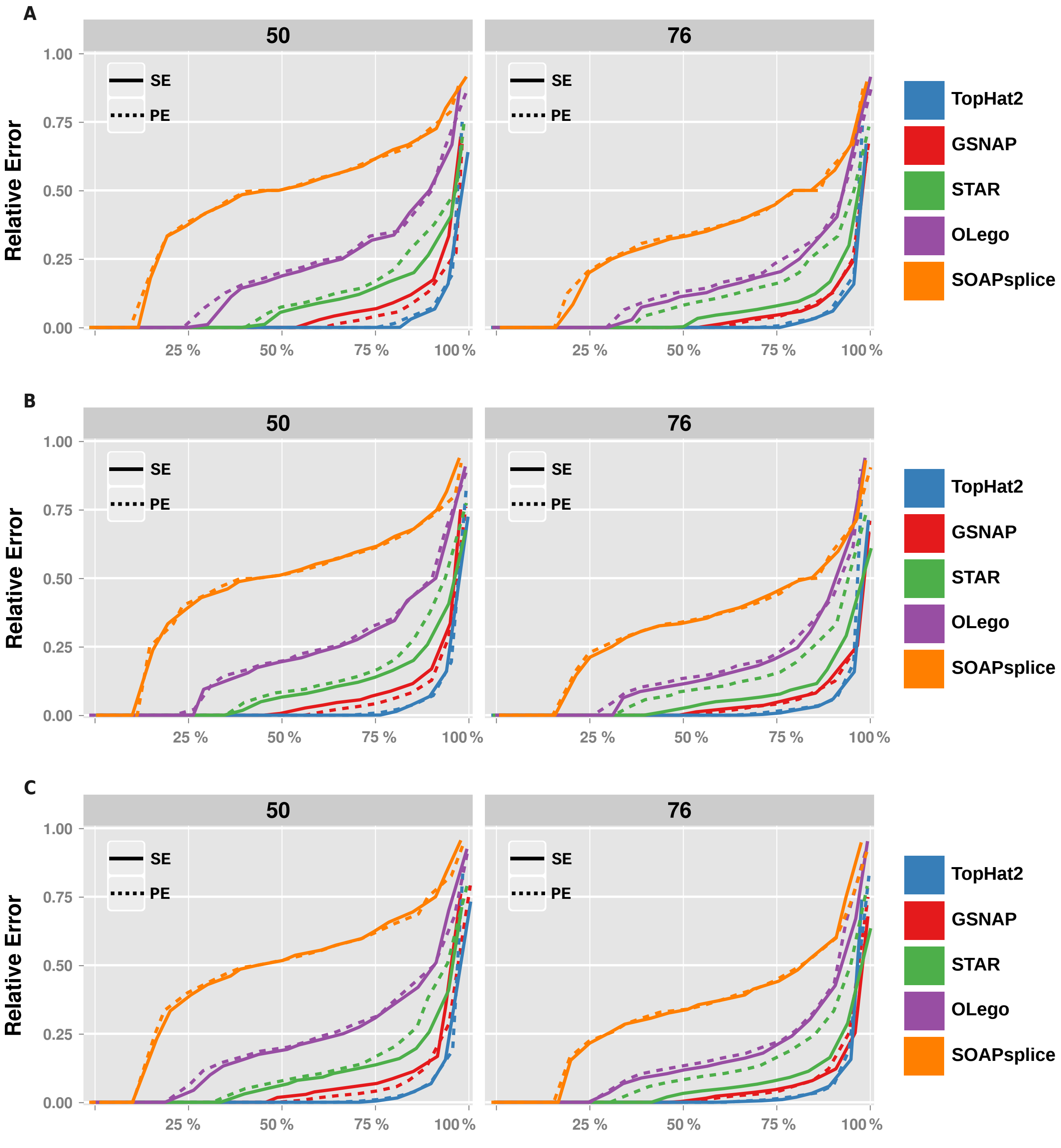

Supplementary Figure 7. Relative quantification error (absolute difference between alignment counts and true read count relative to the true value, *y*-axis) at increasing percentiles (*x*-axis) for each aligner at 8M (**A**), 20M (**B**) and 40M (**C**) reads sequencing depth, all values being averaged over 10 simulated data sets per experimental condition. 50 and 76 bp reads are represented on separate panels, single-end (SE) and paired-end (PE) reads with, respectively, continuous and dashed lines.

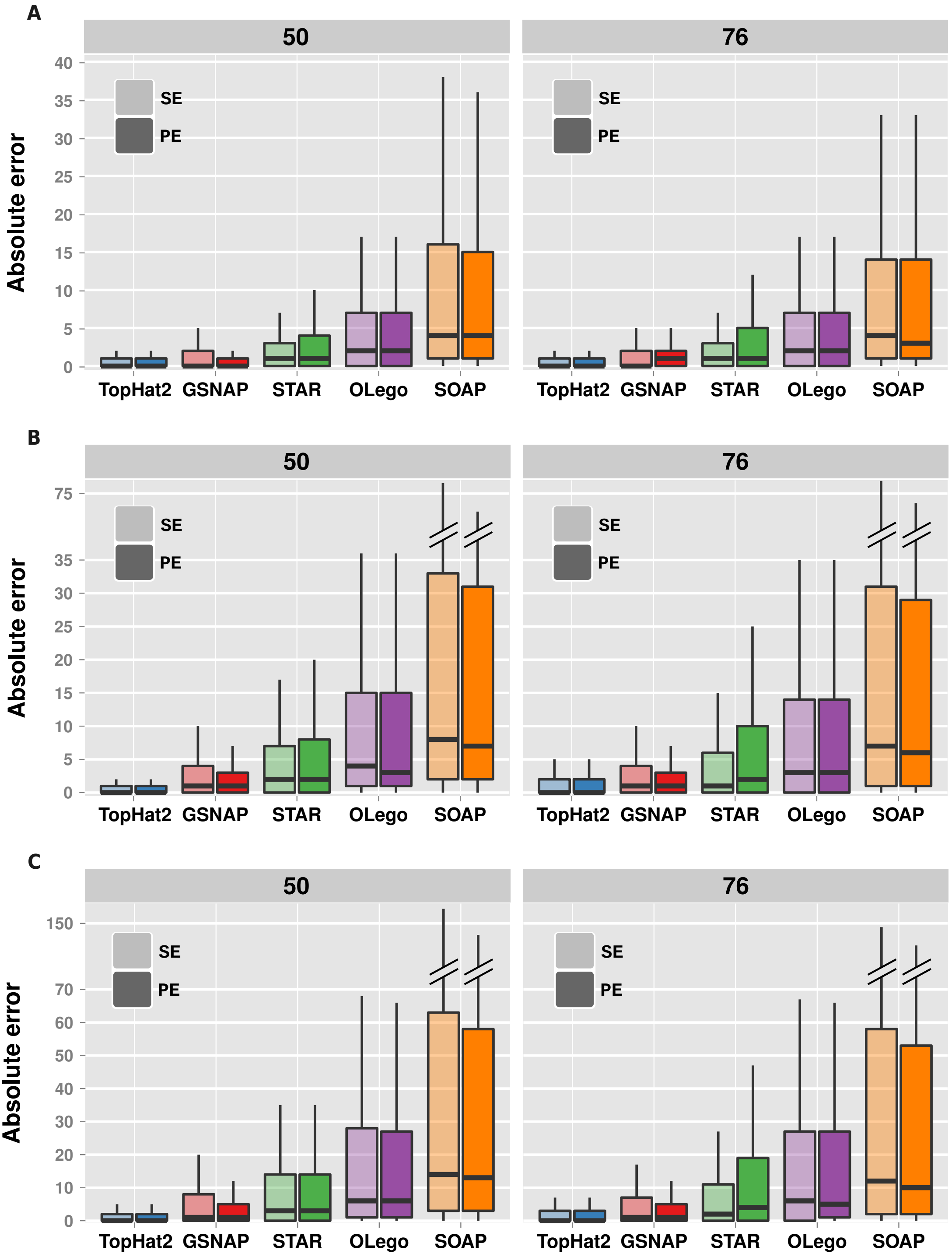

Supplementary Figure 8. Absolute quantification error (absolute difference between alignment counts and true read counts, *y*-axis) in a box plot representation encompassing all 10 simulated data sets, at 8M (**A**), 20M (**B**) and 40M (**C**) reads sequencing depth. 50 and 76 bp reads represented on separate panels, single-end (SE) and paired-end (PE) with distinct fill gradients.

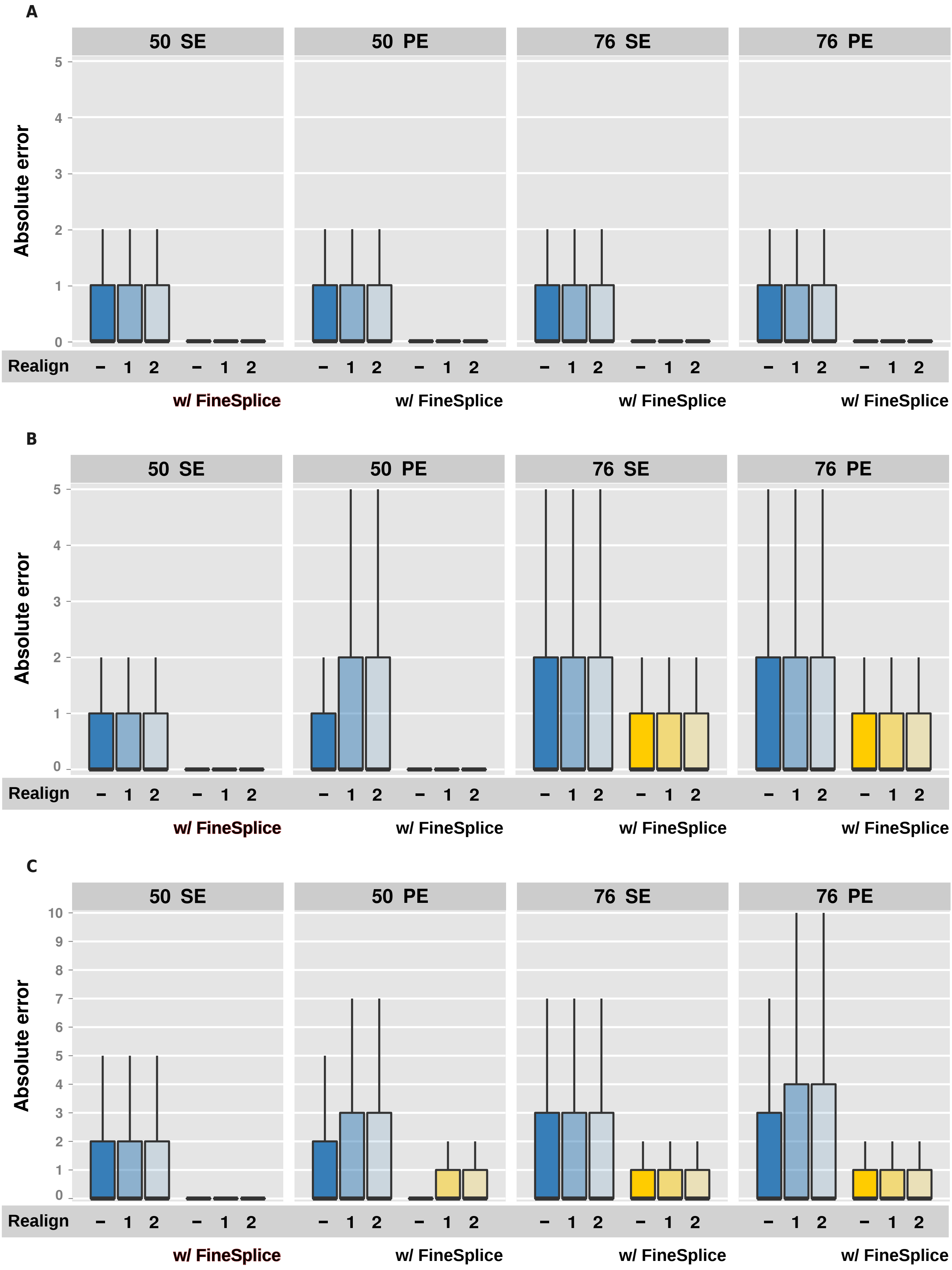

Supplementary Figure 9. TopHat2 absolute quantification error before (blue bars) and after filtering with FineSplice (yellow bars) in a box plot representation (cf. Supplementary Figure 4). Different simulation settings are shown on separate panels: 50 and 76 bp read length, single-end (SE) and paired-end (PE) library, at 8M (**A**), 20M (**B**) and 40M (**C**) reads sequencing depth. Each bar corresponds to different TopHat2 alignment options, either default (-), or with realignment of ambiguously mapping multi-exon reads, allowing up to 1 or 2 mismatches in read segments alignment.

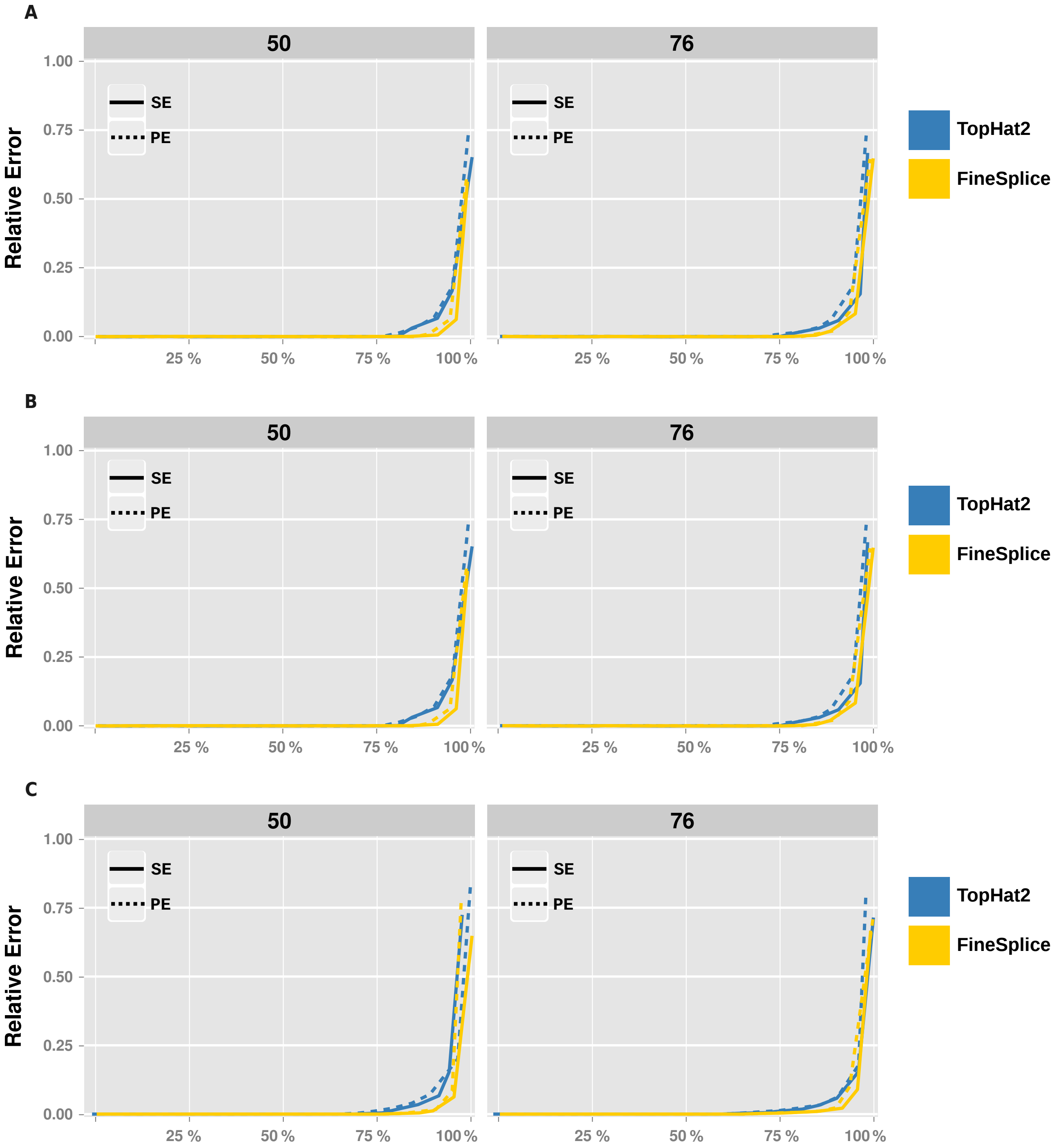

Supplementary Figure 10. TopHat2 relative quantification error percentiles (cf. Supplementary Figure 3), with and without FineSplice at 8M (**A**), 20M (**B**) and 40M (**C**) reads sequencing depth. Different simulation settings on separate panels: 50 and 76 bp read length, single-end (SE) and paired-end (PE) library.

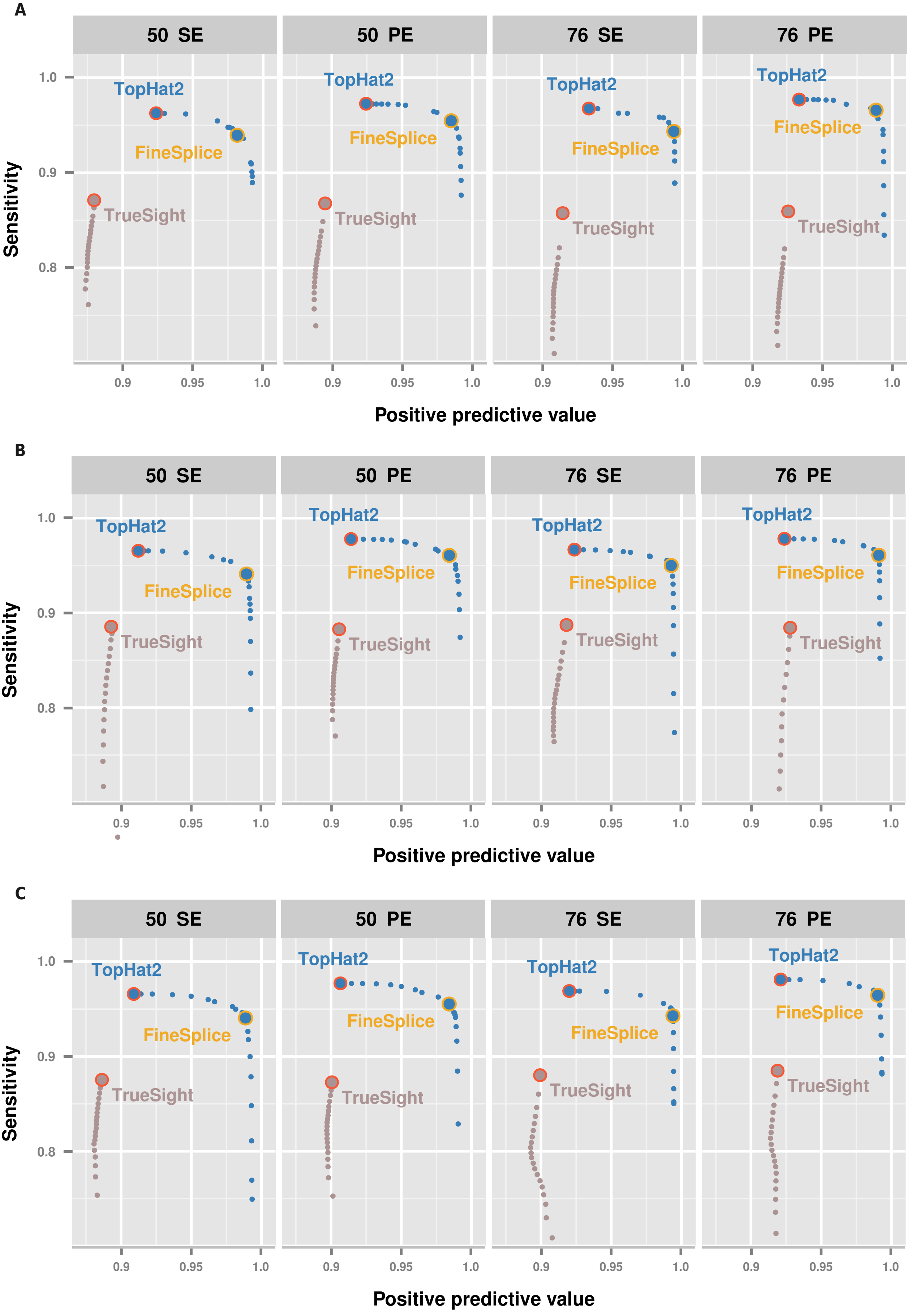

Supplementary Figure 11. Comparison of FineSplice and TrueSight detection performance in simulated data. Sensitivity (*y*-axis) and positive predictive value (*x*-axis) of inferred junctions evaluated at increasing thresholds for the respective score (posterior probabilitiy), with larger dots (outlined) corresponding to the default behaviour of each algorithm (no threshold for TrueSight and TopHat2 alone, 0.5 for TopHat2 w/ FineSplice). Panels correspond to different simulation setups, at 8M (**A**), 20M (**B**) and 40M (**C**) reads sequencing depth.

A

	PPV (mean ± SD)		Sn (mean ± SD)		F$_1$ (mean ± SD)	
TopHat2	0.949	± 0.003	0.923	± 0.002	0.936	± 0.002
FineSplice	0.989	± 0.001	0.918	± 0.003	0.952	± 0.003
GSNAP	0.694	± 0.004	0.998	± 0.001	0.819	± 0.005
STAR	0.825	± 0.020	0.992	± 0.001	0.901	± 0.008
OLego	0.891	± 0.003	0.956	± 0.012	0.922	± 0.009
SOAPsplice	0.873	± 0.002	0.949	± 0.015	0.909	± 0.012
TrueSight	0.986	± 0.001	0.671	± 0.004	0.799	± 0.003

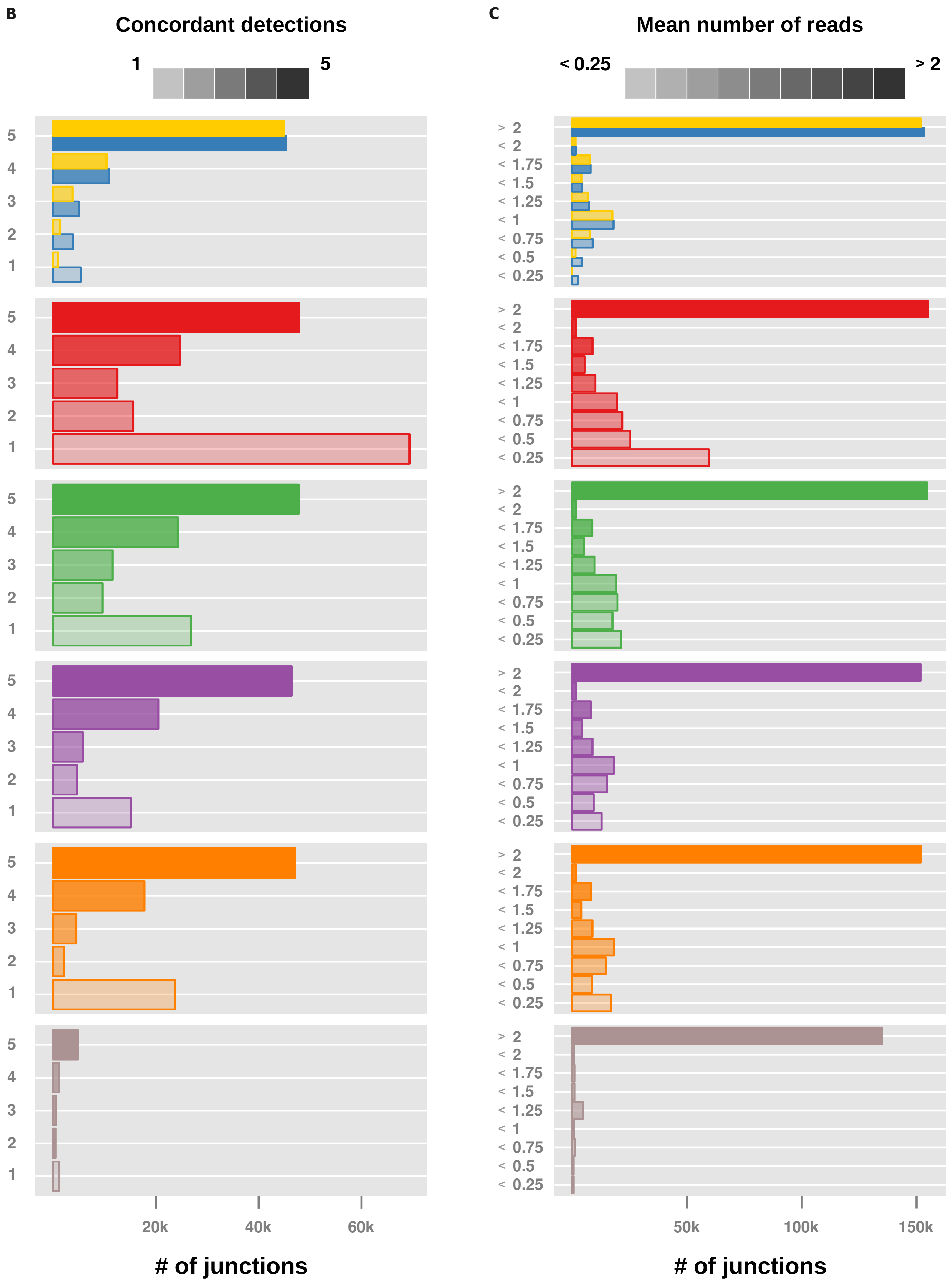

Supplementary Figure 12. Splice junction detection performance in high-quality experimental data (human dataset comprising three high-depth, paired-end sequencing runs at 76 bp read length). Pseudo sensitivity and pseudo precision metrics, along with the corresponding F1 scores, have been evaluated by regarding as true those junctions with a median number of overlapping reads over all alignments greater than 0 (table **A**, mean ± standard deviation). The bar plots show the number of concordant detections (**B**) and the mean read count across all alignments (**C**) for all junctions detected by each method (on separate panels) or accepted by FineSplice (top panel).

A

	PPV (mean ± SD)		Sn (mean ± SD)		F_1 (mean ± SD)	
TopHat2	**0.919**	± 0.003	**0.985**	± 0.001	**0.951**	± 0.001
FineSplice	**0.956**	± 0.001	**0.982**	± 0.001	**0.970**	± 0.000
GSNAP	**0.798**	± 0.010	**0.992**	± 0.000	**0.884**	± 0.006
STAR	**0.928**	± 0.001	**0.979**	± 0.000	**0.952**	± 0.001
OLego	**0.867**	± 0.001	**0.902**	± 0.005	**0.884**	± 0.001
SOAPsplice	**0.962**	± 0.005	**0.727**	± 0.016	**0.828**	± 0.010
TrueSight	**0.984**	± 0.000	**0.562**	± 0.003	**0.715**	± 0.024

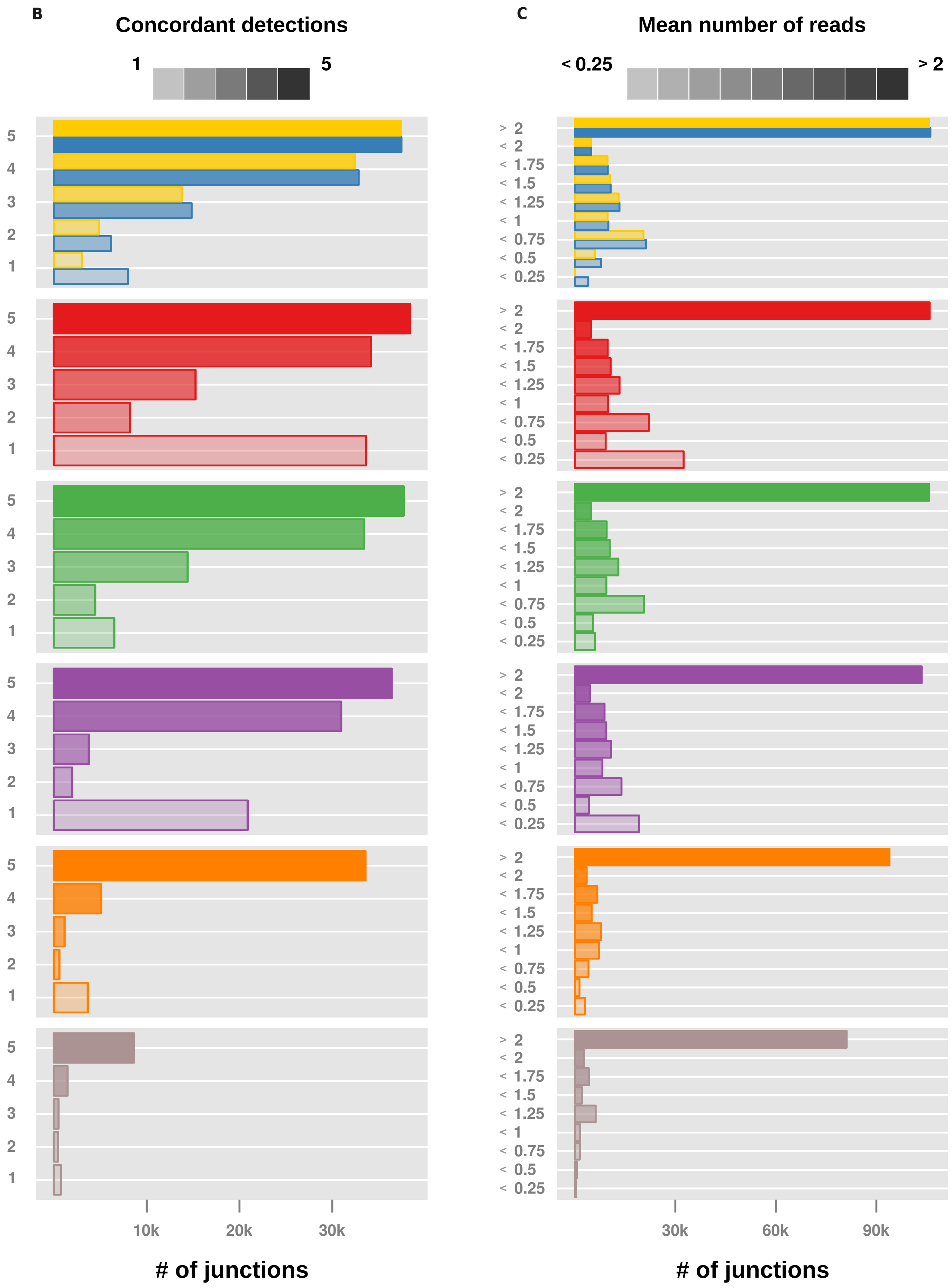

Supplementary Figure 13. Splice junction detection performance in low-quality experimental data (human dataset comprising two low-depth, paired-end sequencing runs at 45 bp read length). Pseudo sensitivity and pseudo precision metrics, along with the corresponding F1 scores, have been computed by regarding as true those junctions with a median number of overlapping reads over all alignments greater than 0 (table **A**, mean ± standard deviation). The bar plots show the number of concordant detections (**B**) and the mean read count across all alignments (**C**) for all junctions detected by each method (on separate panels) or accepted by FineSplice (top panel).

A

	PPV (mean ± SD)		Sn (mean ± SD)		F_1 (mean ± SD)	
TopHat2	**0.939**	± 0.004	**0.944**	± 0.005	**0.942**	± 0.002
FineSplice	**0.978**	± 0.002	**0.941**	± 0.005	**0.960**	± 0.004
GSNAP	**0.958**	± 0.002	**0.972**	± 0.002	**0.965**	± 0.002
STAR	**0.878**	± 0.011	**0.994**	± 0.000	**0.932**	± 0.006
OLego	**0.921**	± 0.005	**0.942**	± 0.064	**0.931**	± 0.004
SOAPsplice	**0.940**	± 0.002	**0.831**	± 0.018	**0.882**	± 0.010
TrueSight	**0.897**	± 0.003	**0.811**	± 0.016	**0.852**	± 0.008

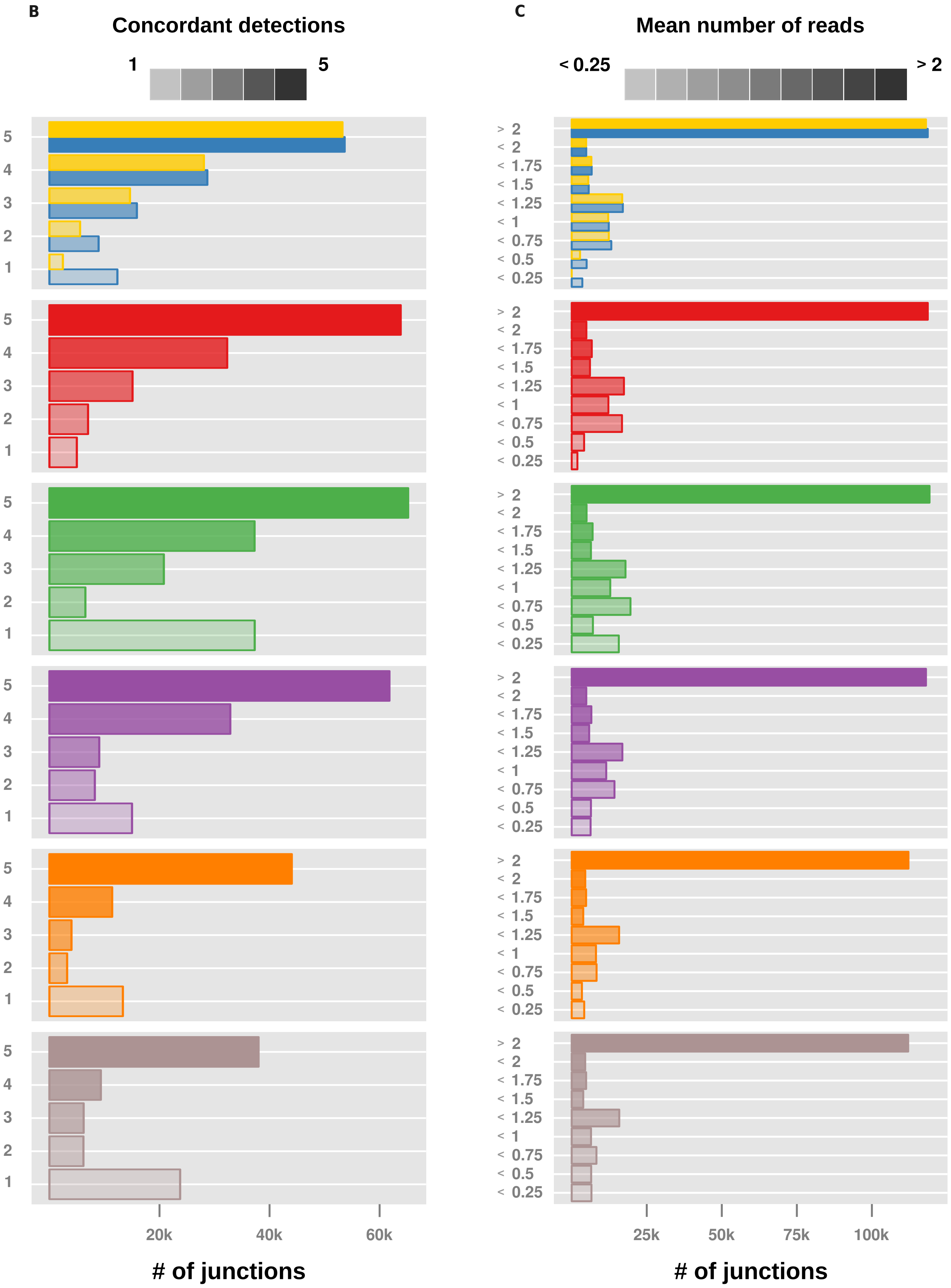

Supplementary Figure 14. Splice junction detection performance in experimental data with poor transcript annotation (pig dataset, comprising three single-end sequencing runs at 51 bp read length). Pseudo sensitivity and pseudo precision metrics, along with the corresponding F1 scores, have been evaluated by regarding as true those junctions with a median number of overlapping reads over all alignments greater than 0 (table **A**, mean ± standard deviation). The bar plots show the number of concordant detections (**B**) and the mean read count across all alignments (**C**) for all junctions detected by each method (on separate panels) or accepted by FineSplice (top panel).

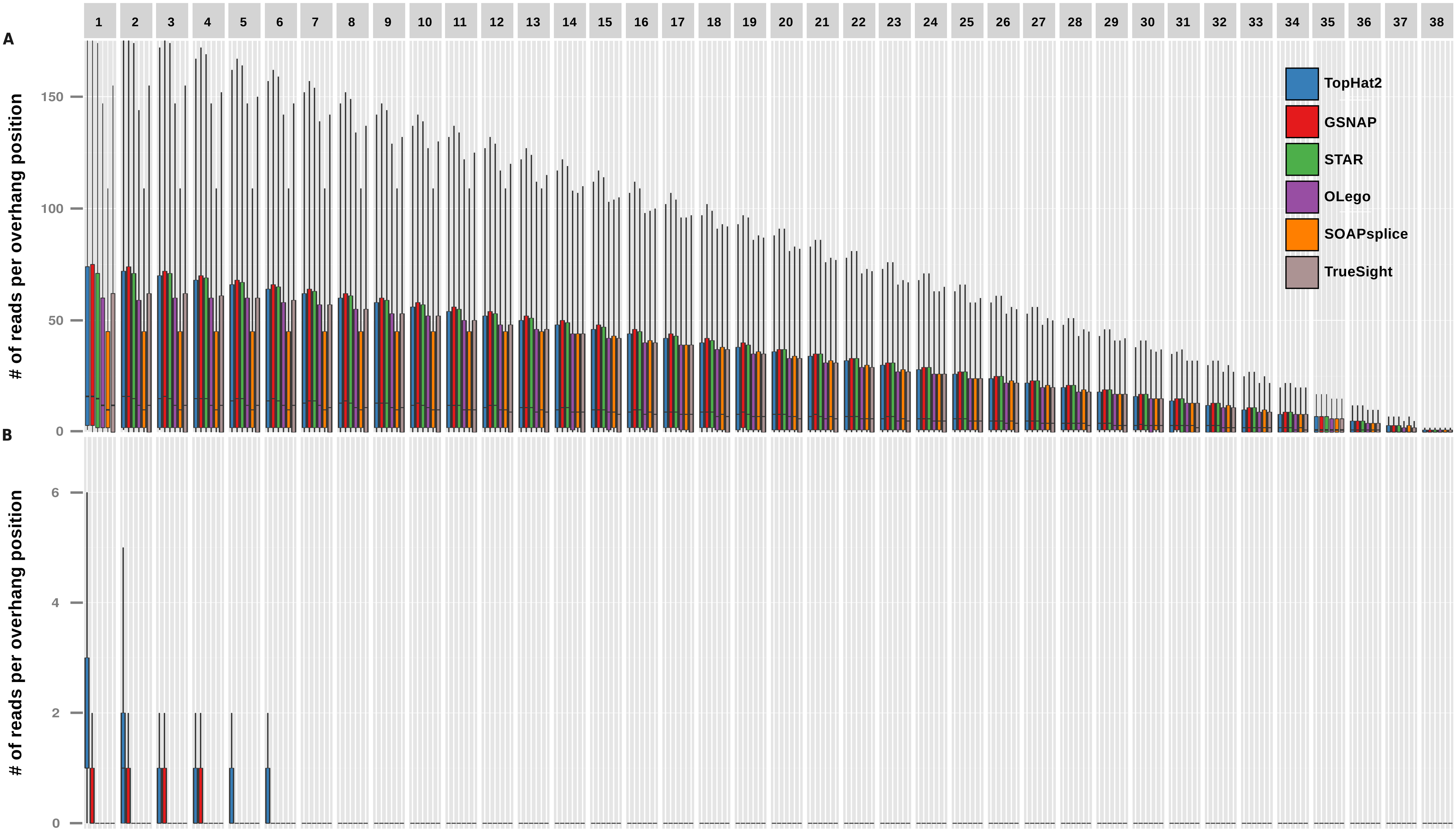

Supplementary Figure 15. Distribution of read overhangs across all alignment methods in experimental high-quality data (human dataset comprising three high-depth, paired-end sequencing runs at 76 bp read length). Read counts per overhang position for splice junctions accepted (**A**) or discarded (**B**) by FineSplice (all sequencing runs) are shown in a box plot representation for each alignment method.

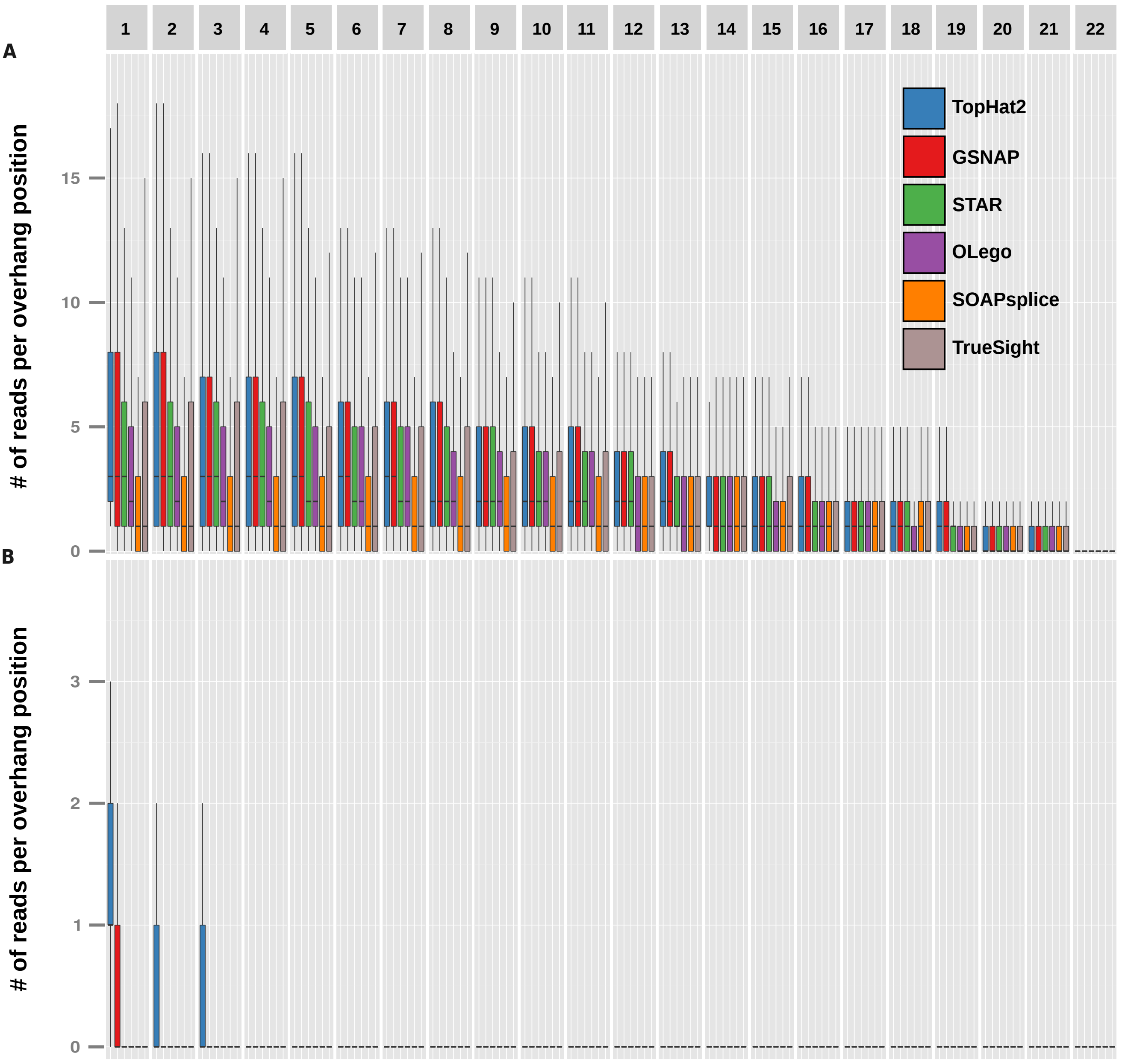

Supplementary Figure 16. Distribution of read overhangs across all alignment methods in experimental low-quality data (human dataset comprising two low-depth, paired-end sequencing runs at 45 bp read length). Read counts per overhang position for splice junctions accepted (**A**) or discarded (**B**) by FineSplice (all sequencing runs) are shown in a box plot representation for each alignment method.

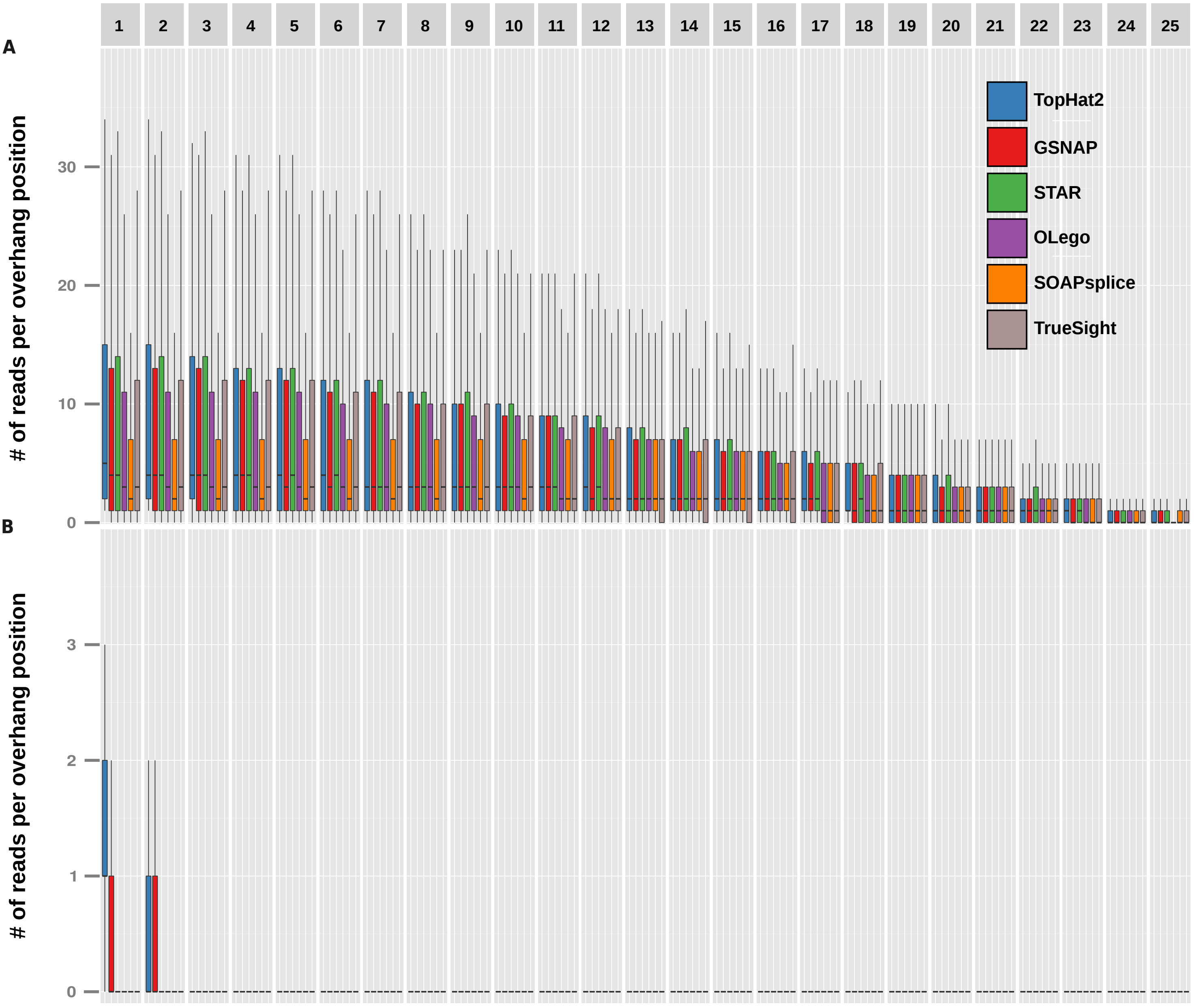

Supplementary Figure 17. Distribution of read overhangs across all alignment methods in experimental data with poor transcript annotation (pig dataset, comprising three single-end sequencing runs at 51 bp read length). Read counts per overhang position for splice junctions accepted (**A**) or discarded (**B**) by FineSplice (all sequencing runs) are shown in a box plot representation for each alignment method.
